# Exploring the Therapeutic Mechanism of Xinbao Pill in Brain Injury After Cardiopulmonary Resuscitation Based on Network Pharmacology, Metabolomics, and Experimental Verification

**DOI:** 10.1111/cns.70297

**Published:** 2025-03-04

**Authors:** Dongli Li, Qihui Wu, Zunjiang Li, Baijian Chen, Xing Sun, Qiqi Wu, Zhenzhu Ding, Linling Liu, Jiansong Fang, Ruifeng Zeng, Yong Gu, Banghan Ding

**Affiliations:** ^1^ The Second Affiliated Hospital of Guangzhou University of Chinese Medicine Guangdong Provincial Hospital of Chinese Medicine Guangzhou China; ^2^ Guangdong Provincial Key Laboratory of Research on Emergency in TCM Guangzhou China; ^3^ Clinical Research Center, Hainan Hospital, Guangdong Provincial Hospital of Chinese Medicine Hainan Medical University Haikou China; ^4^ Science and Technology Innovation Center Guangzhou University of Chinese Medicine Guangzhou China; ^5^ The Second Clinical College of Guangzhou University of Chinese Medicine Guangzhou China

**Keywords:** AMPK, brain injury after cardiopulmonary resuscitation, mitochondrial quality control, Xinbao pill

## Abstract

**Background:**

Post‐cardiopulmonary resuscitation brain injury (PBI) is essentially the cerebral ischemia reperfusion (CIR) injury, which is the main cause of death and long‐term disability in patients with cardiac arrest. So far, there is no treatment for PBI; thus, it is urgent to develop new drugs or therapies for the prevention and treatment of brain injury after cardiopulmonary resuscitation. Although multiple constituent herbs or active ingredients of Xinbao Pill (XBP) have shown neuroprotective effects, whether XBP could play a therapeutic role on PBI is still unknown. This study aimed to illustrate the neuroprotective effect of XBP on PBI and probe the underlying mechanisms.

**Method:**

We first performed the cell and animal experiments to validate the protective effect of XBP on neurological function. We next identified the potential differential metabolites via metabolomics analysis. We further conducted a comprehensive network pharmacology analysis including overlap gene analysis, protein–protein interaction network, and gene–biological process–module function network to preliminarily investigate the specific mechanism of action (MOA) of XBP against PBI. Finally, PCR, MTT, ELISA assay, as well as Western blotting experiments were made to validate our proposed molecular mechanisms.

**Result:**

The in vitro experiment showed that XBP could increase cell viability and ameliorate cell morphological damage in PC12 cells exposed to oxygen–glucose deprivation and reoxygenation (OGD/RO) conditions. The in vivo experiment demonstrated that XBP improved the Neurologic Deficit Score (NDS), lowered the Neuron‐Specific Enolase (NSE) level as well as reversed the typical neuropathological changes in PBI rats, indicating its neuroprotective effect on PBI. Further metabolomics analysis identified 94 differential metabolites after XBP treatment, and multiple metabolites were highly related to CIR. Moreover, network pharmacology results revealed that the therapeutic effect of XBP on PBI may be relevant to mitochondrial quality control (MQC). Mechanistically, XBP could not only promote the expressions of marker proteins including PGC1α, NRF1, TFAM, OPA1, MFN1 as well as MFN2 in mitochondrial biogenesis and mitochondrial fusion but also inhibit those proteins containing DRP1, MFF, FIS1, p62, PINK1, Parkin as well as LC3 in mitochondrial fission and mitophagy. Finally, AMP‐activated protein kinase (AMPK) inhibitor was demonstrated to play a crucial role in regulating MQC.

**Conclusions:**

Our study first determined that XBP might be an underlying anti‐PBI formula, which also deciphered the potential MOAs of XBP against PBI by a network pharmacology approach combined with in vivo and in vitro experimental validation.

## Graphical abstract

A diagram illustrating a network‐based mechanism exploration framework for XBP against brain injury after cardiopulmonary resuscitation. (A) In vivo and in vitro pharmacodynamic confirmation of XBP against PBI. (B) Network pharmacology approach as well as metabolomics analysis to explore the potential mechanisms. (C) Mechanism verification of XBP against PBI.

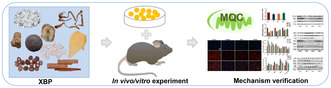

**Abbreviations**
AMPK: AMP‐activated protein kinase
ATP: adenosine triphosphate
BP: biological process
CC: Compound C
CIR: cerebral ischemia reperfusion
CPR: cardiopulmonary resuscitation
DMEM: Dulbecco's modified Eagle's medium
DMSO: dimethyl sulfoxide
HE: Hematoxylin–Eosin
MAP: mean arterial pressure
MMP: mitochondrial membrane potential
MOA: mechanism of action
MQC: mitochondrial quality control
mtDNA: mitochondrial DNA
NDS: neurologic Deficit Score
NSE: neuron‐specific Enolase
OGD/RO: Oxygen–glucose deprivation and reoxygenation
PBI: post‐cardiopulmonary resuscitation brain injury
ROSC: spontaneous circulation
SDH: succinate dehydrogenase
TCM: Traditional Chinese medicine
XBP: Xinbao Pill

## 1 Introduction

Post‐cardiopulmonary resuscitation brain injury (PBI) is induced by initial ischemia resulting from cardiac arrest (CA) and subsequent cerebral reperfusion following cardiopulmonary resuscitation (CPR) [1]. For patients suffering from CA, even if the preliminary resuscitation results are successful, more than 70% of those will die due to the consequences of brain injury after resuscitation [2]. Unfortunately, only by means of targeted temperature management, seizure control and general intensive care management could benefit the neurological recovery of PBI patients [3]. Without direct treatment, PBI causes a serious impact on the quality of life of patients and imposes a heavy burden on their families and social healthcare. Therefore, it is vital to explore new therapeutic strategies to improve the cerebral ischemia–reperfusion (CIR) injury.

The pathophysiology of PBI covers many complex molecular events, including mitochondrial dysfunction, oxidative stress, inflammation, excitotoxicity, and so on [4]. Mitochondria, as the central organelle to produce almost all the energy in the cells, are rich in brain neurons, indicating their vital role in protecting neuronal cells [5]. Meanwhile, increasing literature has proved that mitochondrial dysfunction plays an important part in inducing the death of neuronal cells in PBI [4, 6]. Thus, it is crucial to establish strict quality control mechanisms to ensure healthy mitochondrial homeostasis. The mechanisms of quality control are determined by mitochondrial quality control (MQC) including mitochondrial dynamics, mitochondrial biogenesis, as well as mitophagy [7]. Mitochondrial dynamics, consisting of mitochondrial fusion and fission, allow for the segregation of impaired mitochondria and promote the equipoise of mitochondrial components, including mitochondrial DNA (mtDNA), metabolites, and proteins. Mitochondrial biogenesis is considered the division and growth of pre‐existing mitochondria to change the quality and quantity, which is performed to meet the increasing energy demands of cells [8]. Mitophagy is in charge of degrading and recycling the impaired mitochondria. All these MQC mechanisms can guarantee the best function of mitochondria and protect their structure, further improving neurological functions.

A growing number of literatures demonstrate that traditional Chinese medicine (TCM) exerts an important effect on CIR injury [9, 10]. Xinbao Pill (XBP) is a Chinese herb prescription consisting of *Panax ginseng* C.A.Mey. (root and rhizome), *Datura metel* L. (*Solanaceae*), *Panax notoginseng* (*Burkill*) *F.H.Chen* (*Araliaceae*), *Cervi Cornu Pantotrichum* (*the young horn of Cervus Nippon Temminck*), *Moschus* (*the dried preputial secretion of Moschus berezovskii*, *M. sifanicus*, *or M. moschiferus*), *Cinnamomum verum J. Presl* (*Lauraceae*), *Aconitum carmichaeli Debx* (*Ranunculaceae*), *Bufonis Venenum* (*the venomous glands of Bufo bufo gargarizans Cantor*) as well as *Borneolum Syntheticum* [11]. It was widely demonstrated to treat cardiovascular diseases including chronic heart failure, myocardial ischemia–reperfusion injury, sinus syndrome caused by sinus insufficiency, and so on [11]. Moreover, multiple pieces of evidence have also revealed that the components of XBP perform definite neuroprotection on CIR injury. For example, Ginsenoside Re, Rg3, and Rg1 were found to have neuroprotective effects against CIR injury by inhibiting calcium overload, oxidative stress, as well as neuroinflammation [12], enhancing the activities of SOD and GPx, and stabilizing mitochondria [13]. *Panax notoginseng* and Notoginsenoside R1 ameliorated ischemia reperfusion injury via inhibiting the TLR4/MyD88/NF‐κB signaling pathway and altering microglia polarization [14, 15]. However, the protective effect of XBP on PBI is still ambiguous. Therefore, it is significant to probe the underlying mechanism of XBP against PBI.

In this study, we integrated the network pharmacology approach, metabolomics, as well as experimental validations to reveal the therapeutic mechanism of XBP against PBI. Specifically, we first performed the in vivo and in vitro experiments to confirm the pharmacodynamical protective effect of XBP on PBI. We next conducted a comprehensive network pharmacology analysis, as well as metabolomics, to preliminarily investigate the potential mechanisms of XBP against PBI. Moreover, we utilized multiple experimental methods, including PCR, MTT assay, and Western blotting experiments, to further validate the proposed molecular mechanisms. Finally, an AMPK inhibitor was used to illuminate the mediating role of AMPK in MQC.

## 2 Materials and Methods

### 2.1 Cell Culture

PC12 cells were obtained from the Cell Bank of Shanghai Institute of Biochemistry & Cell Biology at the Chinese Academy of Sciences (Shanghai, China) and cultured in Dulbecco's modified Eagle's medium (DMEM) mixed with 1% penicillin, 1% streptomycin, as well as 10% fetal bovine serum (Gibco BRL, USA). Cells were maintained in an incubator with 5% CO_2_ at 37°C.

### 2.2 Oxygen–Glucose Deprivation and Reoxygenation and Drug Treatment

PC12 cells were exposed to oxygen–glucose deprivation and reoxygenation (OGD/RO) conditions to mimic PBI in vitro [16]. The cells were cultured with glucose‐free DMEM in a three‐gas incubator (1% O_2_, 95% N_2_, 5% CO_2_) at 37°C for 2 h to imitate oxygen–glucose deprivation. At the onset of reperfusion, high‐glucose DMEM containing different concentrations of XBP (0, 250, 500, 1000 μg/mL, Guangdong Xinbao Pharm‐tech Co. Ltd., China) substituted for the glucose‐free DMEM, and the PC12 cells were moved to a stable environment with 5% CO_2_ for 24 h. For the preparation of XBP, we dissolved the XBP powder with ddH_2_O (deionized distilled water) via ultrasonic treatment twice for 30 min at a concentration of 10 mg/mL and then centrifuged at 17320 × *g* for 15 min to extract the supernatant. The precipitate was dissolved with an equal volume of ddH_2_O and sonicated for 30 min twice. Next, it was centrifuged, and the supernatant was extracted as described above. Finally, the two supernatants were combined and concentrated to a concentration of 20 g/L. After concentration, we dissolved the extract with DMEM and filtered it to arrange the required dosage of medication.

### 2.3 Cell Viability Assay

The MTT assay was performed to detect cell viability. PC12 cells were seeded into 96‐well plates at a density of 5000 cells per well and cultured in medium for 24 h. Subsequently, the OGD/RO model and XBP administration were performed. MTT with a concentration of 0.5 mg/mL was added to the medium for 4 h at 37°C, and then 150 μL dimethyl sulfoxide (DMSO) was used to replace the medium. The absorbance was measured at 570 nm with a Microplate reader.

### 2.4 Cell Morphology Observation

The cell morphology was observed by an inverted microscope (Olympus, Tokyo, Japan). Cells at a density of 1.5 × 10^5^ cells/well were plated into six‐well plates. After the OGD/RO model construction and drug administration, cell morphology observation was implemented.

### 2.5 Mitochondrial Membrane Potential (MMP) Assay

MMP was measured by the Mitochondrial Membrane Potential Assay Kit with TMRE (Beyotime Biotechnology, China). At the designed time point of the experiment, PC12 cells were washed with PBS and then incubated with the 1 × TMRE staining solution at 37°C for 20 min. After that, the cells were washed twice with DMEM, stained with Hoechst33342 for 10 min, and the fluorescent images were captured by the DMI8 fluorescence microscope (Leica Microsystems, Wetzlar, Germany).

### 2.6 Mitochondrial Morphology Observation

To inspect the mitochondrial morphology, mitochondria were marked with Mito‐Tracker Red following the instructions of the Mito‐Tracker Red CMXRos kit (Beyotime Biotechnology, China). At the designed time point of the experiment, the cells were incubated with the Mito‐Tracker Red CMXRos solution at 37°C for 20 min after PBS washing. Subsequently, after washing the cells with DMEM twice and staining the cells with Hoechst33342 for 10 min, the cells were washed twice with DMEM again and shot by the SP8 confocal microscope (Leica Microsystems, Wetzlar, Germany).

### 2.7 Animals

Male Sprague–Dawley (SD) rats (330–380 g) were purchased from Hunan SJA Laboratory Animal Co. Ltd. (Hunan, China). All rats were housed in a specific pathogen‐free facility with a 12/12 h light/dark cycle with free access to water and standard chow. All animal experiments were adhered to the principles and guidelines of the National Institutes of Health Guide for the Care and Use of Laboratory Animals and were approved by the Guangdong Provincial Hospital of Traditional Chinese Medicine Laboratory Animal Ethics Committee (Approval Number 2020006).

### 2.8 CA/PCR Model and Drug Treatment

The CA/CPR model was constructed by clamping the endotracheal tube to achieve asphyxia in the rats, which were fasted for 12 h before the experiments. Rats were anesthetized with an intraperitoneal injection of pentobarbital sodium (45 mg/kg) and the hair on the anterior chest, left chest wall, neck, and bilateral inguinal region has been shaved off. Then, a 14‐gauge cannula (Abbocath‐T, USA) was intubated in the trachea, and a 23‐gauge polyethylene catheter (Abbocath‐T, USA) was selected for left femoral artery intubation to monitor mean arterial pressure (MAP). The hemodynamic data were measured and recorded continuously by a computer‐based collection system (WinDaq acquisition system, USA). After instrumentation, we started to clamp the endotracheal tube to induce CA, which was defined as the decrease in MAP below 20 mmHg and the disappearance of arterial pulse. At the end of 5 min of CA, CPR was initiated by mechanical ventilation with 100% O_2_ and precordial compression (PC) at a rate of 250 compressions per min. Simultaneously, epinephrine (0.02 mg/kg) was injected through the femoral artery at 2 min intervals until the return of spontaneous circulation (ROSC) which was characterized by a MAP ≥ 60 mmHg and the appearance of regular supraventricular rhythm lasting at least 5 min. What's more, when ventricular fibrillation occurred, defibrillation with a 2 J biphasic waveform would be performed immediately. Mechanical ventilation and PC would be stopped if the rats failed to ROSC after 10 min of CPR.

All rats were randomly divided into the following groups: (1) Sham group (Sham, *n* = 20), performed the same procedure as other groups and treated with the same volume of saline but without inducing CA/CPR; (2) Post‐cardiopulmonary resuscitation brain injury group (PBI, *n* = 20), treated with the same volume of saline; (3) Post‐cardiopulmonary resuscitation brain injury + low‐dose XBP group (XBP‐L, *n* = 20), treated with XBP 80 mg/kg [17]; (4) Post‐cardiopulmonary resuscitation brain injury + high‐dose XBP group (XBP‐H, *n* = 20), treated with XBP 120 mg/kg [17]. XBP or saline was administered for 7 days before CA and 3 days after ROSC. For drug administration, we dissolved XBP powder in 0.9% saline and prepared the drug solution based on the weight of the rats.

### 2.9 Neurologic Deficit Score

Level of overall performance, brainstem function, muscle strength, algesia, motor performance, behavior, and seizures were assessed based on the method of Neurologic Deficit Score (NDS) [18]. NDS (0 = brain death, 80 = no observed neurologic deficit) was evaluated and graded by two researchers unwitting of the group at 24, 48, and 72 h after ROSC.

### 2.10 Measurement of Neuron‐Specific Enolase (NSE) Level, Adenosine Triphosphate (ATP) Level, Succinate Dehydrogenase (SDH) Level, and Mitochondrial Respiratory Chain Complex I–IV

For cell extracts, they were first centrifuged to obtain the lower layer of precipitated cells. Subsequently, the collected cells were homogenized in ice‐cold double‐distilled water, and the cell suspension was heated to 100°C for 10 min. For animal samples, all rats were anesthetized with pentobarbital sodium (45 mg/kg) after 72 h NDS. The venous blood was collected from the abdominal vein, stewed, and centrifuged at 1000 × *g* for 20 min. Then, the serum was used to measure the NSE level. Next, the rats were sacrificed, and the cortex tissues were rapidly dissected from the brains and stored at −80°C for animal assays. The ATP level of cells or cortex tissues was detected in accordance with the manufacturer's instructions of the ATP assay kit (Nanjing Jiancheng Bioengineering Institute, Nanjing, China). The level of SDH and mitochondrial respiratory chain complexes I—IV of mitochondria extracted from the cortex was measured using the Elisa assay kits (Jianglai Biotechnology, Shanghai, China).

### 2.11 Hematoxylin–Eosin (HE) Staining

HE staining was used for observing the neuronal morphology in the cortex. The rats were intracardially perfused with 0.9% saline and 4% paraformaldehyde. Brains were obtained and immersed in 4% paraformaldehyde, and the coronal sections embedded in paraffin were cut into 5 μm thick sections for HE staining. Next, the HE staining was conducted according to the manufacturer's instructions, and the slides were analyzed by a light microscope (Leica Microsystems, Wetzlar, Germany).

### 2.12 Western Blotting Analysis

Western blotting was conducted as previously reported [19]. Anti‐Hsp60 (1: 1000, Abcam), anti‐ATPB (1: 1000, Abcam), anti‐VDAC1 (1: 1000, Abcam), anti‐PGC‐1 alpha (1: 1000, Abcam), anti‐NRF1 (1: 1000, Abcam), anti‐TFAM (1: 1000, CST), anti‐DRP1 (1: 1000, Abcam), anti‐MFF (1: 1000, Abcam), anti‐FIS1 (1: 1000, Affinity), anti‐OPA1 (1: 1000, Abcam), anti‐Mitofusin 1 (1: 1000, Affinity), anti‐Mitofusin 2 (1: 1000, Affinity), anti‐SQSTM1/p62 (1: 1000, Abcam), anti‐PINK1 (1: 1000, Abcam), anti‐Parkin (1: 1000, Abcam), anti‐LC3 (1: 1000, Abcam), anti‐AMPK alpha (1: 1000, Affinity), and anti‐Phospho‐AMPK alpha (Thr172) (1: 1000, Affinity) were the primary antibodies. Immunoreactivity was analyzed and quantified by Image J software (Bio‐Rad Laboratories, California, USA).

### 2.13 Mitochondrial DNA and AMPK Copy Number

DNAiso Reagent (Takara Bio, Japan) was used to extract the mitochondrial DNA (mtDNA) from PC12 cells or cortex tissues according to the manufacturer's protocol. Trizol reagent (Invitrogen, USA) was adopted to extract total RNA for measuring AMPK copy number. Reverse transcription was performed using PrimeScriptTM RT Master Mix (Perfect Real Time) (Takara Bio, Japan) and reverse transcription PCR was quantified via utilizing TB Green Premix Ex TaqTM II (Tli RNaseH Plus) (Takara Bio, Japan). Target gene expression was normalized to β‐actin expression (internal control) using the 2^−ΔΔ*C*^_T_ method. All primer sequences (5′‐> 3′) were listed as follows: MT‐NADH1, AACCCTCTCCCTTACACT (forward), TTGATGCTCATCCTGATC (reverse); AMPK, CGTTCTATTGCCACTCTGC (forward), CATAGGAGGGGTCTTCAGG (reverse); β‐actin, GAGAGGGAAATCGTGCGT (forward), GGAGGAAGAGGATGCGG (reverse).

### 2.14 Metabolomics Analysis

Serum sample was added to 1200 μL of internal standard extract solution containing 70% methanol, vortexed for 3 min, and centrifuged at 13680 × *g* at 4°C for 10 min to obtain the supernatant. The obtained supernatant (50 μL) was redissolved in internal standard extract solution including 20% acetonitrile and methanol for LC–MS/MS analysis. Chromatographic separation was performed at a flow rate of 0.35 mL/min at 40°C and an injection volume of 4 μL. The mobile phase comprised solvent A (water with 0.1% formic acid) and solvent B (acetonitrile with 0.1% formic acid). The gradient elution program was as below: 0 min 5% B, 0–9 min 95% B, 9–10 min 95% B, 10.0–11.1 min 5% B, 11.1–14 min 5% B. Qualitative analysis of metabolite was conducted based on a self‐built database called the metware database (MWDB), and metabolite quantification was performed through multireaction monitoring mode of triple quadrupole mass spectrometry, and the data were analyzed by Analyst 1.6.3 [20]. Principal Component Analysis (PCA) was executed to obtain a preliminary understanding of the overall metabolic differences between each group of samples and the magnitude of variability among samples within the group. Orthogonal Partial Least Squares Discrimination Analysis (OPLS‐DA) was used to observe the classifications for different groups. Significantly regulated metabolites between groups were determined by VIP ≥ 1 and absolute log_2_FC (fold change) ≥ 1. VIP values were extracted from OPLS‐DA results, which also contained score plots and permutation plots, generated using the R package MetaboAnalystR.

### 2.15 Integration associated with Genes of Mitochondrial Dysfunction

We searched the keyword “mitochondrial dysfunction” in three authoritative databases, including DisGenet database (https://www.disgenet.org/), GeneCards database (https://previous.malacards.org/pages/info), and the Open Targets database (https://www.opentargets.org/). After removing the duplicates, 178 genes associated with mitochondrial dysfunction (Table S1) were obtained. Networks in this study were generated via cytoscape (v3.2.1, http://www.cytoscape.org/). The detailed integration process of the protein–protein interactions can be found in previous literatures [21].

### 2.16 Data Analysis

All experimental data were presented as mean ± SD and analyzed by SPSS 23.0 statistical software. One‐way analysis of variance (ANOVA), followed by Tukey's multiple comparison test was performed for group comparisons. The analysis of NDS was conducted based on a generalized linear model.

## 3 Results

### 3.1 XBP Improved Neurological Function

#### 3.1.1 XBP Increased Cell Viability and Ameliorated Cell Morphological Damage in PC12 Cells Under OGD/RO Exposure

**FIGURE 1:**
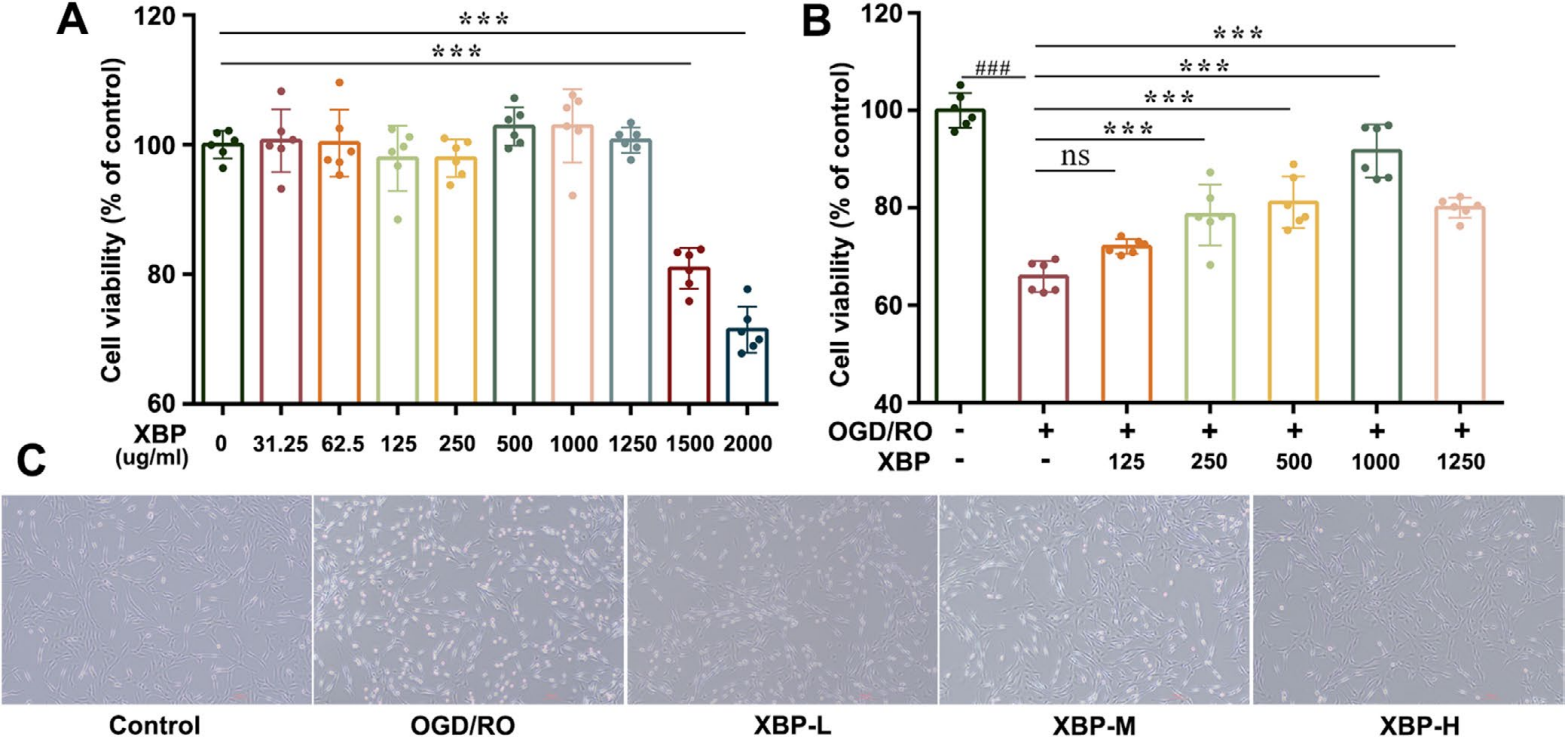
XBP improved neurological function in PC12 cells under OGD/RO exposure. The cytotoxic effect (A) and protective effect (B) of XBP were detected by MTT assay. (C) Images of cell morphology were shown. Data are expressed as mean ± SD *N* = 6. One‐way ANOVA with Turkey's post hoc test (A, B). ###*p* < 0.001 versus the Control group. ***p < 0.001 versus the OGD/RO group. Control, Control group; OGD/RO, OGD/RO group; XBP‐H, OGD/RO + high‐dose Xinbao Pill group; XBP‐L, OGD/RO + low‐dose Xinbao Pill group; XBP‐M, OGD/RO + middle‐dose Xinbao Pill group.

MTT assay and cellular morphological observation were performed to evaluate the neuroprotective effect of XBP on PBI in vitro. Firstly, the cytotoxicity of XBP in PC12 cells was determined by MTT assay. We found that XBP did not induce the cytotoxic effect when the PC12 cells were incubated with XBP at the dosages from 31.25 to 1250 μg/mL (Figure 1A) for 24 h. Meanwhile, we estimated the positive effect of XBP on PC12 cell viability, which was exposed to OGD/RO conditions. As shown in Figure 1B, XBP (250, 500, 1000 μg/mL, chosen as the low, medium and high doses for the experiment) enhanced the survival rate in a dose‐dependent manner (*p* < 0.05). In addition, cellular morphological observation revealed that XBP could reverse the morphological alterations, including cell shape, detachment, and the shrinkage of cell bodies induced by OGD/RO conditions (Figure 1C). These results indicated that XBP existed as a neuroprotective effect on PC12 cells under OGD/RO exposure.

#### 3.1.2 XBP Ameliorated Neurological Function in PBI Rats

**FIGURE 2:**
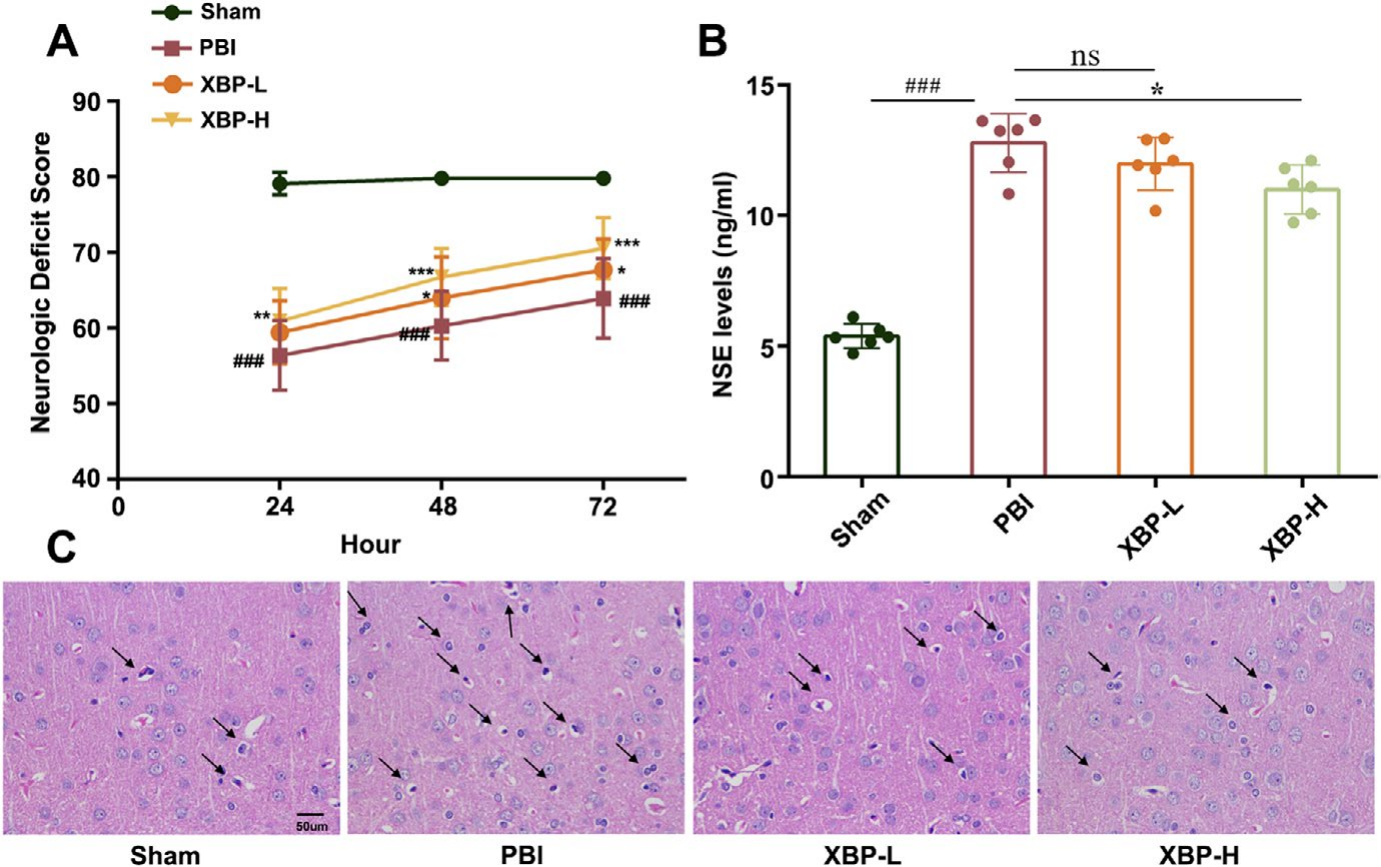
XBP ameliorates neurological function in PBI rats. (A) NDS at 24, 48, and 72 h were recorded (Sham: *N* = 10; PBI: *N* = 13; XBP‐L: *N* = 14; XBP‐H: *N* = 16, generalize linear model). (B) NSE level in serum was detected by Elisa assay kit (*N* = 6, one‐way ANOVA with Turkey's post hoc test). (C) Images of HE staining in cortex were shown. Data are expressed as mean ± SD. ###*p* < 0.001 versus the Sham group. *p < 0.05, **p < 0.01, ***p < 0.001 versus the PBI group. PBI, Post‐cardiopulmonary resuscitation brain injury group; Sham, Sham group; XBP‐H, Post‐cardiopulmonary resuscitation brain injury + high‐dose Xinbao Pill group; XBP‐L, Post‐cardiopulmonary resuscitation brain injury + low‐dose Xinbao Pill group.

NDS, NSE level, and HE staining were applied to assess whether XBP could improve neurological deficit. NDS, with a higher score representing milder brain injury, was performed to evaluate the effect of XBP on recovery from brain injury at 24, 48, and 72 h after ROSC. As shown in Figure 2A, NDSs in the PBI group and XBP groups increased with the extension of time, and the NDSs in the XBP‐H groups were remarkably higher than those in the PBI groups (*p* < 0.01), suggesting a better effect in ameliorating brain injury. Since NSE level in serum was the indicator of brain injury, we next examined the effect of XBP on the neurological deficit by measuring NSE level. The PBI rats displayed remarkably high NSE levels, whereas XBP‐H treatment significantly decreased the NSE level in the PBI rats (*p* < 0.05) (Figure 2B). Moreover, HE staining was performed to explore the effect of XBP on the neuronal morphology in the cortex. Typical neuropathological changes, including cell shrinkage, cell body shrinkage, as well as hyperchromatic nuclei in the PBI rats, could be reversed by XBP (Figure 2C). Overall, these results suggested the protective effect of XBP on neurological function in PBI rats.

### 3.2 Differential Metabolites in Serum Between the XBP Group and the Control Group Identified by the Metabolomics Analysis

**FIGURE 3:**
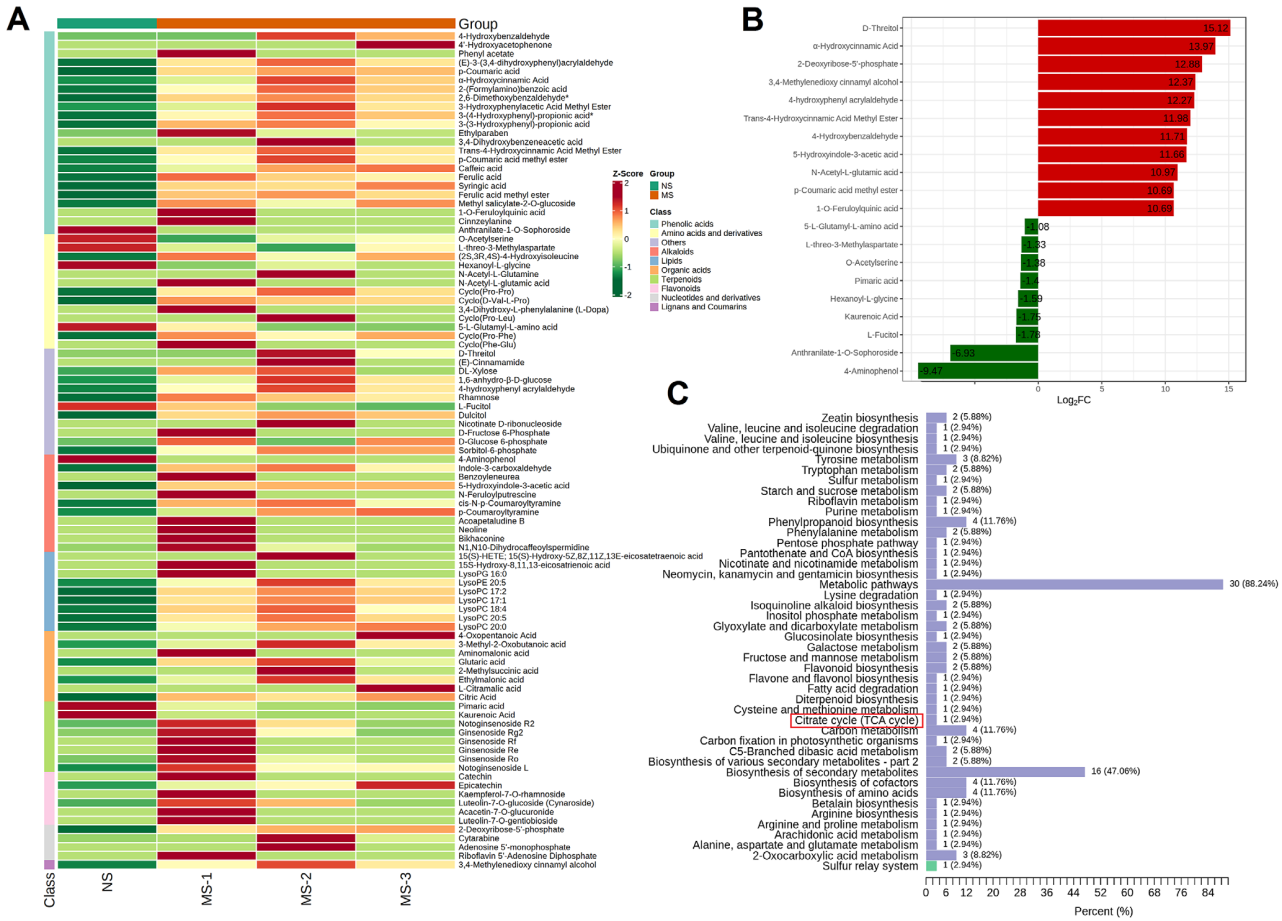
Metabolomics analysis of the XBP group and the control group. (A) Differential metabolite classes in serum between the XBP group and the control group. (B) Top 10 upregulated and downregulated metabolites. (C) KEGG classification of identified metabolites.

To determine the differential metabolites of XBP on PBI, metabolomics analysis in serum samples from rats was performed. In total, 94 differential metabolites were identified by the metabolomics analysis, which could be integrated into 10 categories (Figure 3A). Among them, the most representative category was the phenolic acids (*n* = 23), followed by the amino acids and derivatives (*n* = 13) and others (*n* = 12). Moreover, the top 10 upregulated and downregulated differentiated metabolites in the XBP group were also highlighted, indicating that XBP might partly adjust the disordered metabolites in CIR to normal level (Figure 3B). The upregulated differentiated metabolites are D‐threitol (Fold change [FC] = 15.12), α‐hydroxycinnamic acid (FC = 13.97), 2‐deoxyribose‐5′‐phosphate (FC = 12.88), 2‐deoxyribose‐5′‐phosphate (FC = 12.37), 4‐hydroxyphenyl acrylaldehyde (FC = 12.27), trans‐4‐hydroxycinnamic acid methyl ester (FC = 11.98), 4‐hydroxybenzaldehyde (FC = 11.71), 5‐hydroxyindole‐3‐acetic acid (FC = 11.66), N‐acetyl‐L‐glutamic acid (FC = 10.97), and p‐Coumaric acid methyl ester (FC = 10.69), while the downregulated differentiated metabolites are 1‐O‐Feruloylquinic acid (FC = 10.69), 5‐L‐Glutamyl‐L‐amino acid (FC = −1.08), L‐threo‐3‐Methylaspartate (FC = −1.33), O‐Acetylserine (FC = −1.38), Pimaric acid (FC = −1.4), Hexanoyl‐L‐glycine (FC = −1.59), Kaurenoic Acid (FC = −1.75), L‐Fucitol (FC = −1.78), Anthranilate‐1‐O‐Sophoroside (FC = −6.93), and 4‐Aminophenol (FC = −9.47). To preliminarily explore the potential mechanism of XBP against PBI, we integrated the KEGG classification of identified metabolites (Figure 3C) and found that they were involved with multiple KEGG classifications, such as the citrate cycle (TCA cycle), metabolic pathways, and biosynthesis of secondary metabolites.

### 3.3 Network Pharmacology Analysis

**FIGURE 4:**
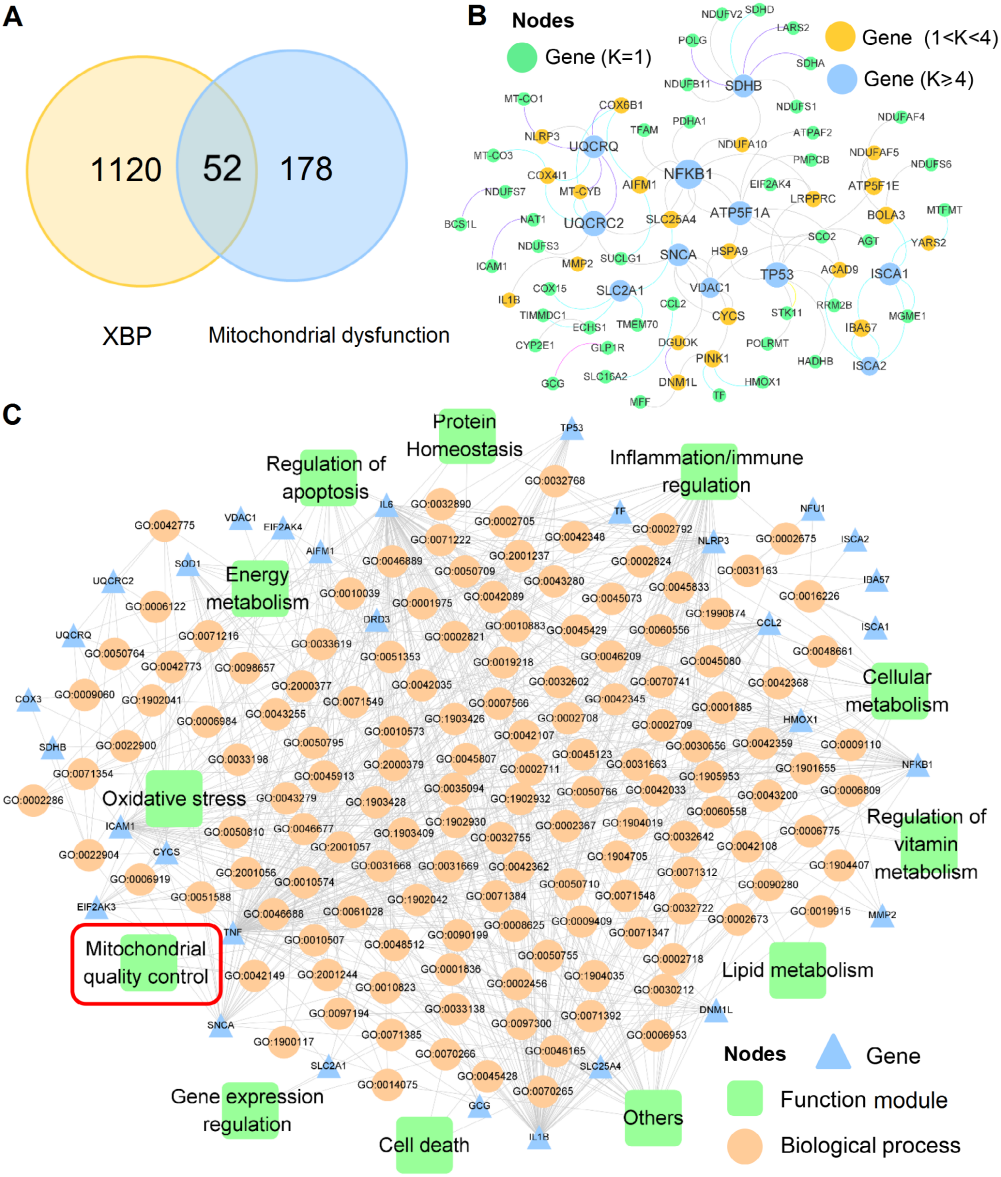
Network pharmacology analysis of the hub genes. (A) Overlap analysis between the mitochondrial dysfunction genes and target proteins that Xinbao Pill acted on. (B) Protein–protein interaction network. (C) Gene–biological process–function module network.

In this part, we firstly integrated the ingredients of XBP and performed the cluster analysis (Figure S1A,B). We next constructed the drug‐target network of XBP, which comprises 2644 DTIs connecting 225 compounds with 1172 genes (Figure S1C). The detailed integration process of the drug–target network could be found in our previous study. Since our KEGG classification of identified metabolites has indicated the importance of the TCA cycle, which is distributed in mitochondria, and mitochondrial function plays a vital role in CIR, we inferred that the therapeutic effect of XBP on CIR might be related to mitochondrial dysfunction. To validate our assumption, we firstly collected the genes associated with mitochondrial dysfunction (see Section 2) and performed an overlapped analysis between the mitochondrial dysfunction genes and target proteins XBP (1172 genes) acted on to identify the hub genes. As shown in Figure 4A, there are a total of 52 overlapped genes, such as NLRP3, NFKB1, and ICAM1. Multiple evidence has indicated their high correlations with CIR. Taking NLRP3 for example, recent research has concluded that NLRP3 inflammasome activation might be a therapeutic target for CIR injury [22, 23]. We next constructed and simplified the PPI network of these genes (Figure 4B), which comprised 99 PPIs and 75 genes.

To decipher the specific mechanism of XBP mitigating CIR, we also performed the biological process (BP) by using the 52 overlapped genes and classified them into different categories according to the literature evidence. As depicted in Figure 4C, these BPs were mainly involved in 12 function modules, including MQC, regulation of apoptosis, energy metabolism, and so forth Indeed, the literature has suggested that these modules (e.g., MQC, neuronal apoptosis) played vital roles in CIR. For instance, CIR was reported to induce neuronal apoptosis in vivo [24], while suppressing apoptosis might reduce CIR [25]. MQC, which contains mitochondrial dynamics (fission and fusion), mitophagy, and mitochondrial biogenesis, and so forth, also serves as a crucial part in CIR. The biophysical and complex processes associated with MQC could protect the mitochondria from injury and weaken the vulnerability of neurons to I/R injury [26] which were consistent with our metabolomic analysis results.

### 3.4 XBP Alleviates Mitochondrial Damage

#### 3.4.1 XBP Affected the Mitochondrial Damage in PC12 Cells Under OGD/RO Exposure

**FIGURE 5:**
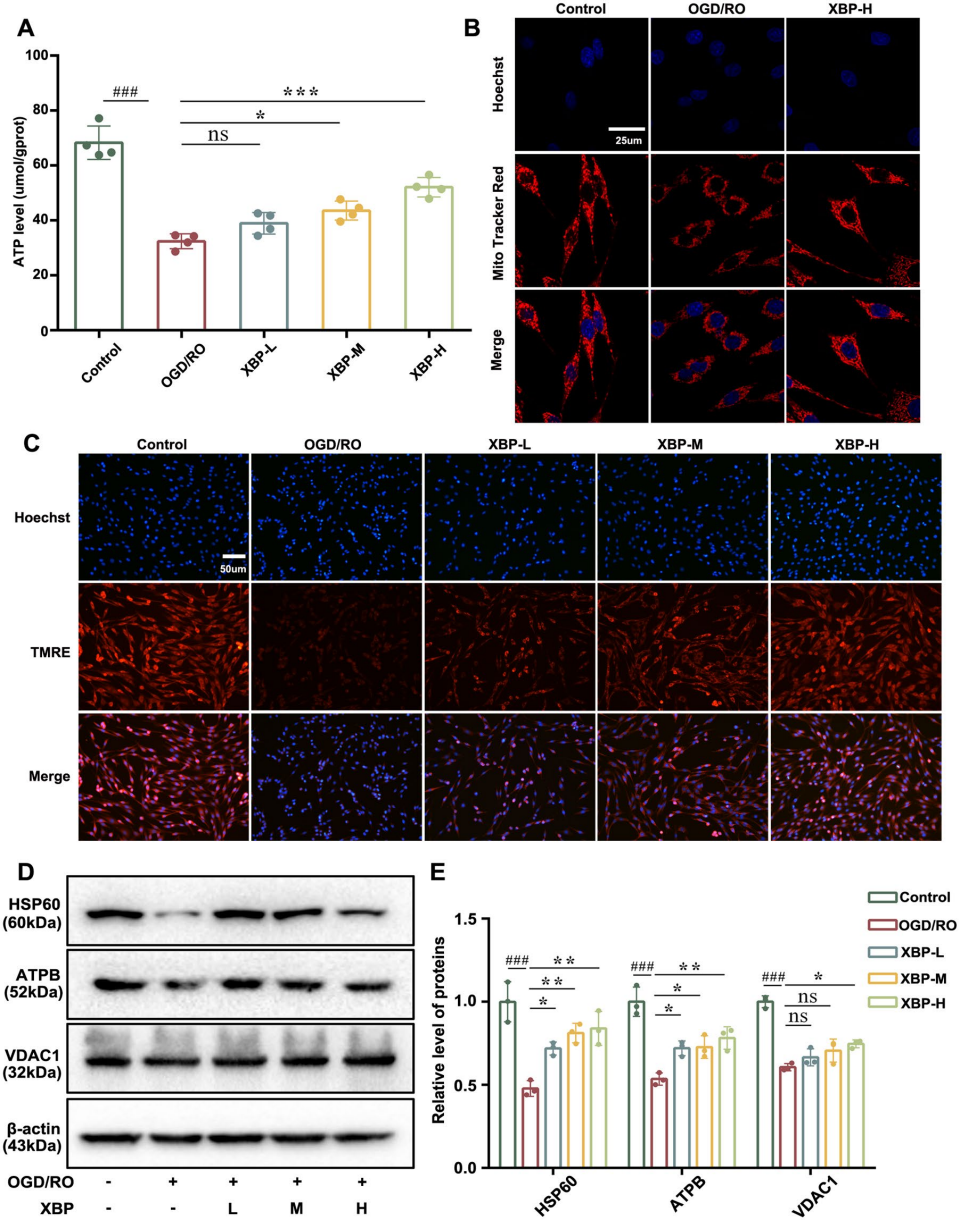
XBP affected the mitochondrial damage in PC12 cells under OGD/RO exposure. (A) ATP level was detected by ATP assay kit (*N* = 4, one‐way ANOVA with Turkey's post hoc test). Images of mitochondrial morphology (B) and MMP (C) were showed. (D, E) The expression level of HSP60, ATPB (one‐way ANOVA with Turkey's post hoc test) and VDAC1 (one‐way ANOVA with Dunnett's T3 post hoc test) were measured by Western blotting (*N* = 3). Data are expressed as mean ± SD. ###*p* < 0.001 versus the Control group. *p < 0.05, **p < 0.01, ***p < 0.001 versus the OGD/RO group. Control, Control group; OGD/RO, OGD/RO group; XBP‐H, OGD/RO + high‐dose Xinbao Pill group; XBP‐L, OGD/RO + low‐dose Xinbao Pill group; XBP‐M, OGD/RO + middle‐dose Xinbao Pill group.

As mentioned above, XBP might regulate the MQC to exert therapeutic effects on PBI. Thus, we first evaluated whether XBP positively affected the mitochondrial damage by detecting the ATP level and MMP since the ATP level and MMP are highly related to mitochondrial damage [27]. As shown in Figure 5A, XBP significantly suppressed the decrease of ATP level in PC12 cells induced by OGD/RO. Meanwhile, low‐intensity orange fluorescence indicated the lessening of MMP in PC12 cells after OGD/RO exposure, which could be improved outstandingly by XBP (Figure 5C). Furthermore, mitochondrial morphology results (Figure 5B) showed that the morphology of mitochondria in the control group was elongated, mesh‐shaped as well as surrounded by strong fluorescence. However, OGD/RO exposure damaged most of the mitochondria to punctuate with weaker fluorescence, which could be relieved by XBP treatment. Subsequently, we measured the expressions of mitochondrial proteins including HSP60, ATPB, and VDAC1 by Western blotting analysis. Results indicated that the PC12 cells exposed to OGD/RO downregulated the levels of HSP60, ATPB, and VDAC1, which were restored by XBP treatment to varying degrees (Figure 5D,E). All these data illustrated the protective effect of XBP on mitochondrial damage in OGD/RO‐induced cells.

#### 3.4.2 XBP Protected Against Mitochondrial Damage in PBI Rats

**FIGURE 6:**
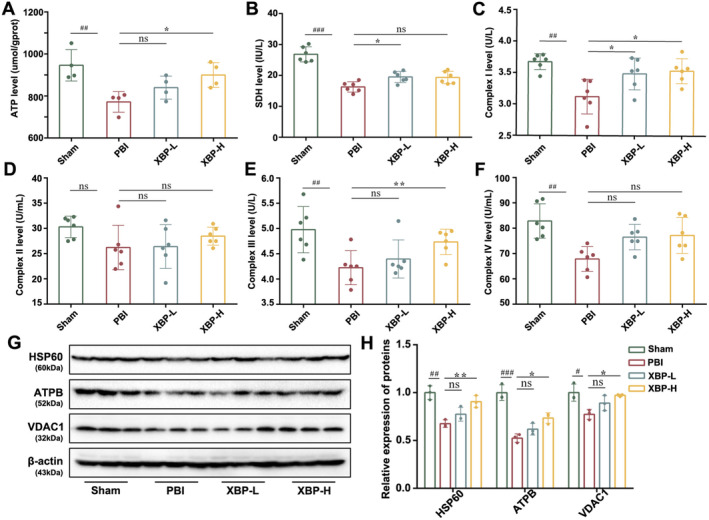
XBP protected against mitochondrial damage in PBI rats. (A) ATP level (*N* = 4) was detected by the ATP assay kit. The levels (B, C, D, E, F) of SDH (*N* = 6), complex I (*N* = 6), complex II (*N* = 6), complex III (*N* = 5) and complex IV (*N* = 6) were detected by the Elisa assay kit. The expressions (*N* = 3) of HSP60, ATPB, and VDAC1 (G, H) were measured by Western blotting. Data are expressed as mean ± SD (one‐way ANOVA with Turkey's post hoc test). #*p* < 0.05, ##*p* < 0.01, ###*p* < 0.001 versus the Sham group. *p < 0.05, **p < 0.01 versus the PBI group. PBI, Post‐cardiopulmonary resuscitation brain injury group; Sham, Sham group; XBP‐H, Post‐cardiopulmonary resuscitation brain injury + high‐dose Xinbao Pill group; XBP‐L, Post‐cardiopulmonary resuscitation brain injury + low‐dose Xinbao Pill group.

To illuminate the positive effect of XBP on PBI in vivo, we detected the ATP level and the key enzyme activities related to mitochondria in PBI rats. The ATP assay showed that XBP could increase the ATP level in PBI rats (Figure 6A). In addition, the activities of crucial enzymes containing SDH and Mitochondrial Respiratory Chain Complex I–IV were examined by ELISA. The activities of SDH, complex I, complex III, and complex IV in the PBI group were decreased; whereas, XBP only enhanced the activities of SDH, complex I, and complex III remarkably, with no significant effect on complex II and complex IV (Figure 6B–F). Furthermore, the expressions of various mitochondrial proteins in the cortex were observed by Western blotting. As shown in Figure 6G,H, the expressions of HSP60, ATPB, and VDAC1 in the PBI group were remarkably lower than those in the Sham group, while XBP promoted the expressions of HSP60, ATPB, as well as VDAC1. Taken together, XBP alleviated mitochondrial damage in rats induced by PBI.

### 3.5 XBP Modulated Mitochondrial Quality Control

To further determine the molecular mechanisms of XBP against PBI, experimental indicators related to MQC, including mitochondrial biogenesis, mitochondrial dynamics, and mitophagy, were examined in PC12 cells under OGD/RO exposure and PBI rats.

#### 3.5.1 Effect of XBP on Mitochondrial Biogenesis

**FIGURE 7:**
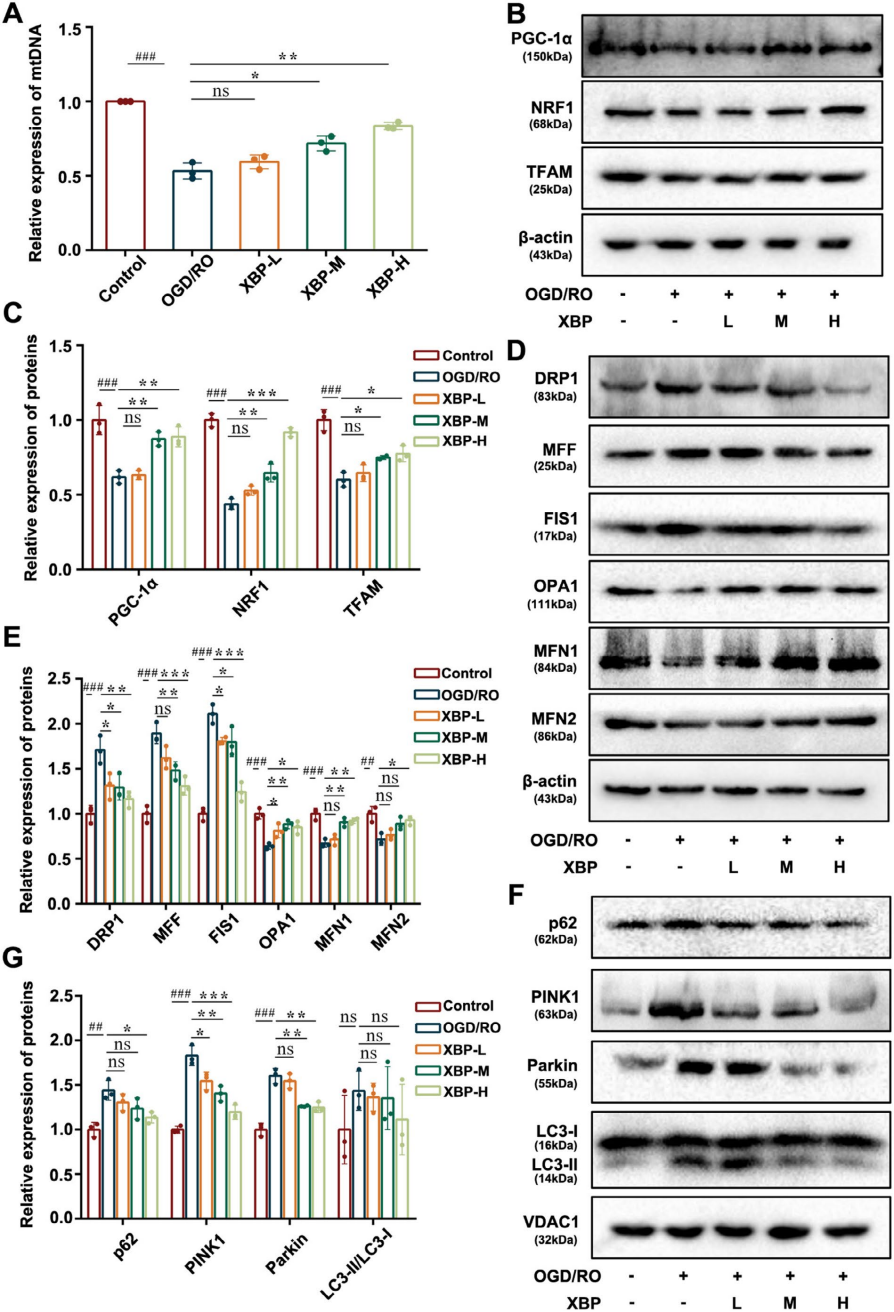
XBP modulated mitochondrial quality control in PC12 cells. (A) Expression of mtDNA was analyzed by PCR. The expression levels of PGC‐1α, NRF1, TFAM (B, C), DRP1, MFF, FIS1, OPA1, MFN1, MFN2 (D, E), p62, PINK1, Parkin, and LC3 (F, G) were measured by Western blotting (*N* = 3, one‐way ANOVA with Turkey's post hoc test). Data are expressed as mean ± SD. ##*p* < 0.01, ###*p* < 0.001 versus the Control group. *p < 0.05, **p < 0.01, ***p < 0.001 versus the OGD/RO group. Control, Control group; OGD/RO, OGD/RO group; XBP‐H, OGD/RO + high‐dose Xinbao Pill group; XBP‐L, OGD/RO + low‐dose Xinbao Pill group; XBP‐M, OGD/RO + middle‐dose Xinbao Pill group.

**FIGURE 8:**
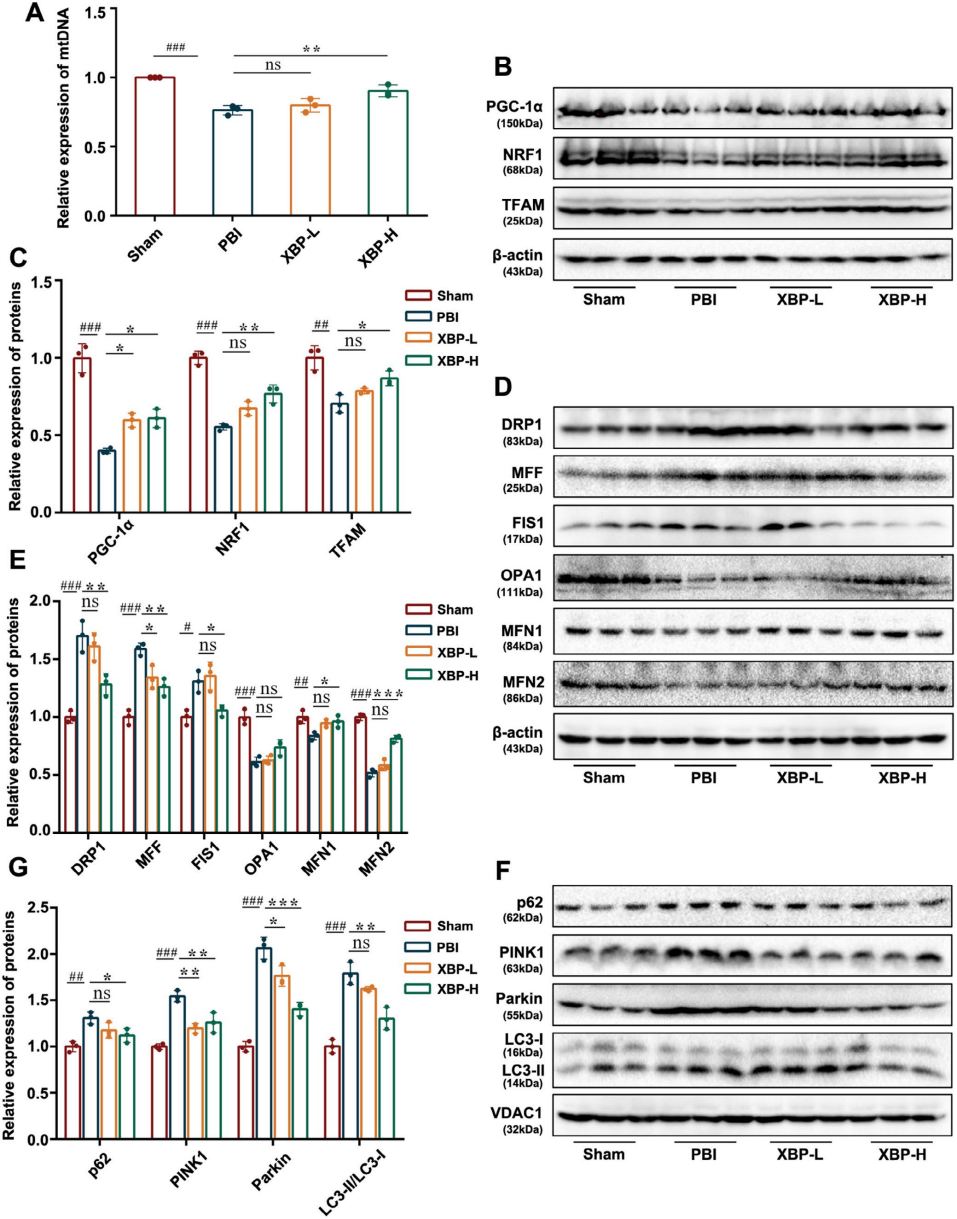
XBP modulated mitochondrial quality control in PBI rats. (A) Expression of mtDNA was analyzed by PCR. The expression level of PGC‐1α, NRF1, TFAM (B, C), DRP1, MFF, FIS1, OPA1, MFN1, MFN2 (D, E), p62, PINK1, Parkin, and LC3 (F, G) were measured by Western blotting (*N* = 3, one‐way ANOVA with Turkey's post hoc test). Data are expressed as mean ± SD. #*p* < 0.05, ##*p* < 0.01, ###*p* < 0.001 versus the Sham group. *p < 0.05, **p < 0.01, ***p < 0.001 versus the PBI group. PBI, Post‐cardiopulmonary resuscitation brain injury group; Sham, Sham group; XBP‐H, Post‐cardiopulmonary resuscitation brain injury + high‐dose Xinbao Pill group; XBP‐L, Post‐cardiopulmonary resuscitation brain injury + low‐dose Xinbao Pill group.

Mitochondrial biogenesis closely regulates the PGC1α/NRF1/TFAM pathway, which could adjust mtDNA copy numbers [28]. As shown in Figure 7A and Figure 8A, the mtDNA copy numbers in PC12 cells exposed to OGD/RO as well as PBI rats were substantially decreased, which could be reversed by XBP treatment. Besides, Western blotting data revealed that intervention with XBP restored the downregulation of PGC1α, NRF1, and TFAM expression in both the OGD/RO induced PC12 cells and PBI rats (Figures 7B,C and 8B,C). These data clarified the beneficial effect of XBP on mitochondrial biogenesis.

#### 3.5.2 Effect of XBP on Mitochondrial Dynamics

Mitochondrial dynamics consists of mitochondrial fission and mitochondrial fusion. Mitochondrial fission is regulated by DRP1, MFF, and FIS1, while mitochondrial fusion is mediated by OPA1, MFN1, and MFN2. To further probe the effect of XBP on mitochondrial dynamics, we investigated the expression changes of these six proteins by Western blotting. We uncovered that (Figures 7D,E and 8D,E), in both PC12 cells under OGD/RO conditions and PBI rats, the expressions of DRP1, MFF, and FIS1 were increased, whereas the levels of MFN1 and MFN2 were obviously decreased. All these changes could be remarkably reversed by XBP. Nevertheless, XBP treatment only promoted the OPA1 level reduced by OGD/RO in PC12 cells. Taken together, these observations demonstrated that XBP could not only stimulate mitochondrial fusion but also inhibit mitochondrial fission.

#### 3.5.3 Effect of XBP on Mitophagy

As previously reported [29] mitophagy can be mediated by LC3‐II through the SQSTM1/p62 pathway and suppressed by the downregulation of Parkin and PINK1. Thus, we detected the protein expression levels of p62, PINK1, Parkin, and LC3 to explore the effect of XBP on mitophagy. Western blotting analysis (Figures 7F,G, and 8F,G) suggested that XBP inhibited the upregulation of p62, PINK1, and Parkin in PC12 cells exposed to OGD/RO and PBI rats. However, the ratio of LC3‐II to LC3‐I could be reduced by XBP treatment only in PBI rats (Figure 8G). The result proved that XBP suppressed mitophagy activation.

### 3.6 AMPK Intermediates the Regulation of XBP on Mitochondrial Quality Control

**FIGURE 9:**
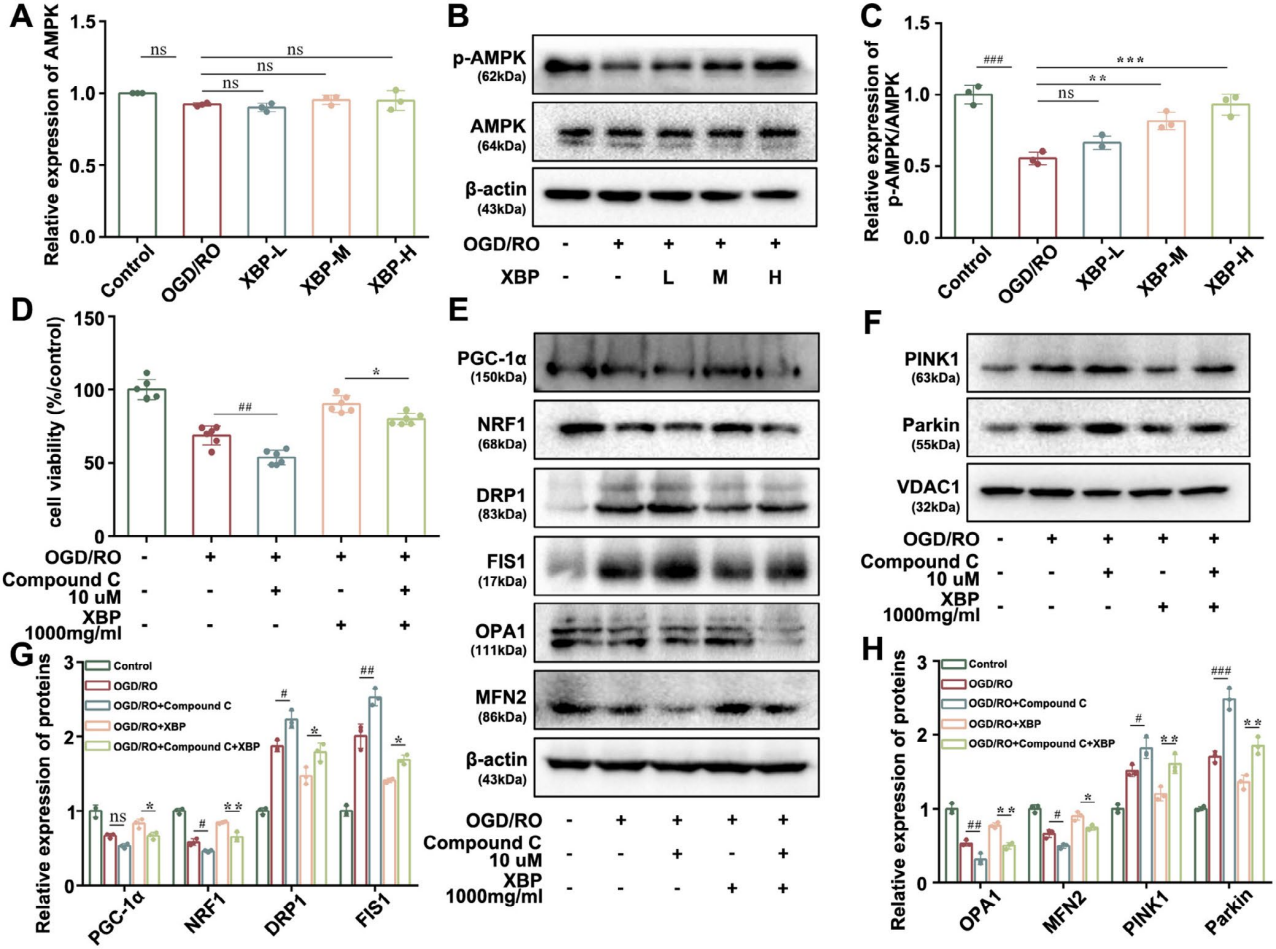
AMPK intermediated the regulation of XBP on mitochondrial quality control in PC12 cells. (A) Expression of AMPK was analyzed by PCR (*N* = 3). The expression levels of p‐AMPK/AMPK (B, C), PGC‐1α, NRF1, DRP1, FIS1, OPA1, MFN2, PINK1, and Parkin (E, F, G, H) were measured by Western blotting (*N* = 3). (D) Cell viability was detected by MTT assay (*N* = 6). Data are expressed as mean ± SD (one‐way ANOVA with Turkey's post hoc test). (A, C) ###*p* < 0.001 versus the Control group. **p < 0.01, ***p < 0.001 versus the OGD/RO group. (D, G, H) #*p* < 0.05, ##*p* < 0.01, ###*p* < 0.001 versus the OGD/RO group. *p < 0.05, **p < 0.01 versus the OGD/RO + XBP group. Control, Control group; OGD/RO, OGD/RO group; XBP‐H, OGD/RO + high‐dose Xinbao Pill group; XBP‐L, OGD/RO + low‐dose Xinbao Pill group; XBP‐M, OGD/RO + middle‐dose Xinbao Pill group.

**FIGURE 10:**
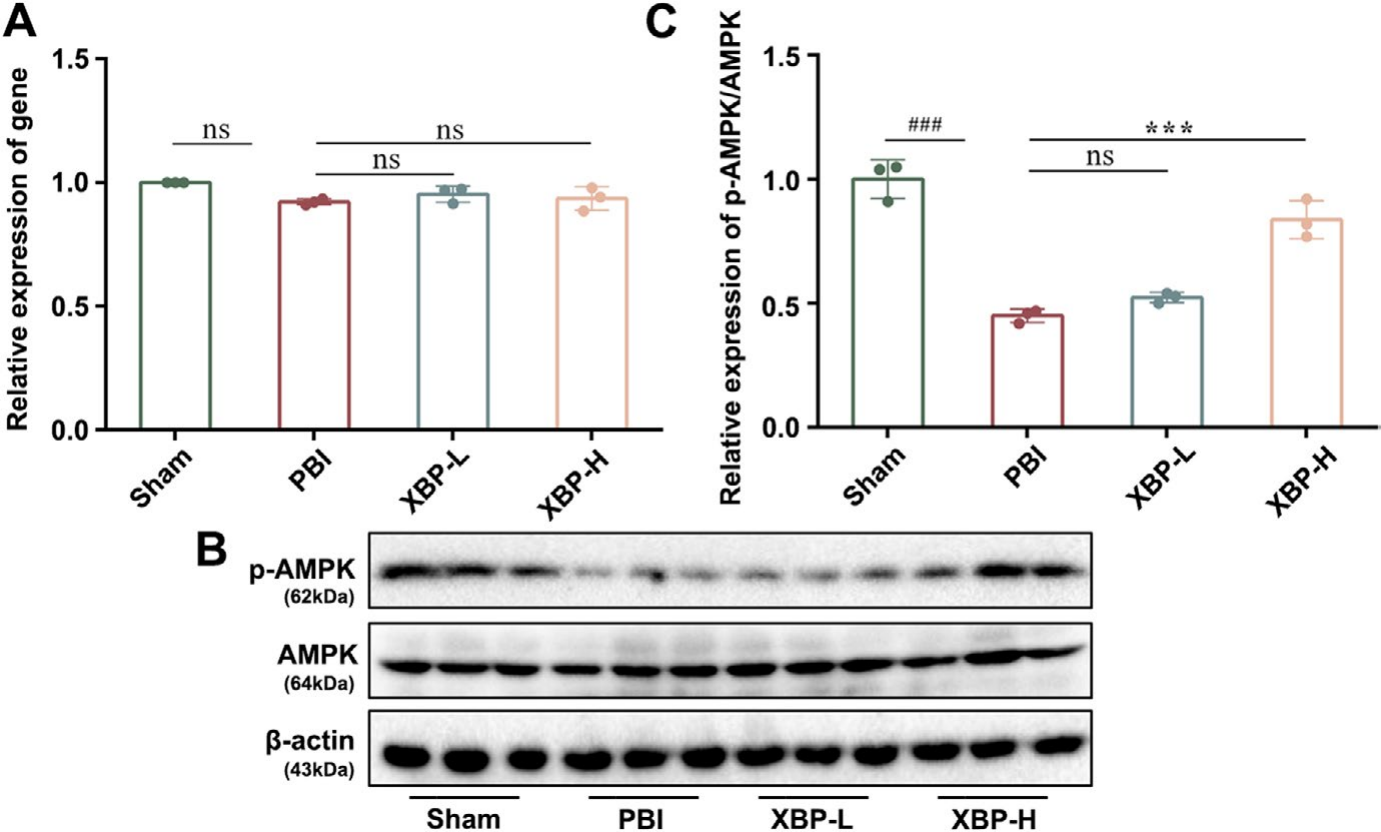
AMPK intermediated the regulation of XBP on mitochondrial quality control in PBI rats. (A) Expression of AMPK was analyzed by PCR. The expression of p‐AMPK/AMPK (B, C) was measured by Western blotting. Data are expressed as mean ± SD (*N* = 3, one‐way ANOVA with Turkey's post hoc test). ###*p* < 0.001 versus the Sham group. ***p < 0.001 versus the PBI group. PBI, Post‐cardiopulmonary resuscitation brain injury group; Sham, Sham group; XBP‐H, Post‐cardiopulmonary resuscitation brain injury + high‐dose Xinbao Pill group; XBP‐L, Post‐cardiopulmonary resuscitation brain injury + low‐dose Xinbao Pill group.

Although the neuroprotective effect of XBP on PBI had been demonstrated, as mentioned above, the potential MOA still required further clarification. AMPK has been acknowledged as an endogenous defensive molecule that responds to various harmful stimuli, such as CIR injury, and plays a key role in regulating MQC [30]. Thus, we first measured the expression levels of AMPK and phosphorylated AMPK (p‐AMPK) in PC12 cells under OGD/RO exposure and PBI rats. RT‐PCR and Western blotting data showed that XBP had no significant effect on the copy number and protein expression of AMPK (Figures 9A,B and 10A,B). Interestingly, the protein expression of p‐AMPK could be increased by XBP, which revealed that XBP promoted the phosphorylation of AMPK (Figures 9B,C and 10B,C). Moreover, Compound C (CC, 10 μM), as an AMPK inhibitor, was applied to PC12 cells exposed to OGD/RO to verify whether AMPK mediated the administration of XBP on MQC. Results implied that compound C eliminated the positive effect of XBP on cell viability and modulated the levels of PGC‐1α, NRF1, OPA1, MFN2, DRP1, FIS1, PINK1, Parkin, as well as VDAC1 to some extent (Figure 9D–H), demonstrating that the adjustment of XBP on MQC was AMPK‐dependent.

## 4 Discussion

CIR injury after cardiopulmonary resuscitation keeps a substantive reason for morbidity and mortality, while there are still no drugs or therapies for it. Increasing studies have demonstrated that traditional Chinese medicine exists a positive effect in ameliorating CIR injury and presents beneficial therapeutic potential [31, 32]. In this study, we found that XBP had a protective effect on PBI both in vitro and in vivo. Subsequently, we explored the MOAs of XBP against PBI by metabolomics analysis as well as a comprehensive network pharmacology analysis, and the results illustrated that the therapeutic effect of XBP on PBI was relevant to MQC. Finally, further experimental validation was performed to confirm our proposed molecular mechanisms.

Increasing evidence has shown that mitochondrial dysfunction was a key event in CIR injury [33]. When mitochondria are damaged, oxidative phosphorylation dysfunction occurs, and ATP production decreases accordingly. The reduced ATP cannot support the transfer of H^+^ from the matrix to outside the inner mitochondrial membrane to counteract the electrochemical gradient, resulting in the disruption and decrease of MMP [27]. Meanwhile, the electron transport via complexes I—III—IV as well as II—III—IV is out of order and impaired, which is manifested as reduced mitochondrial respiratory chain complexes activities and linked with the reduced ATP induced by mitochondrial damage [34]. In this study, the metabolomics data indicated that the mitochondrial dysfunction was related to XBP against PBI. Thus, the levels of ATP, complex I, II, III, IV, and MMP were detected. Our results showed the decrease of ATP, complex I, III, IV levels, and MMP, which corresponded to the literature, demonstrating the pivotal effect of mitochondrial dysfunction in CIR injury. Meanwhile, we also discovered the changes in mitochondrial morphology as well as the decrease in expressions of mitochondrial proteins. Furthermore, the activity of SDH, a key enzyme that connects electron transfer and oxidative phosphorylation, was also reduced, revealing that mitochondria were damaged. However, XBP treatment could restore the above damage to some extent, suggesting that the improvement effect of XBP on PBI might target mitochondria.

MQC is the main control mechanism for regulating mitochondrial morphology, quality, quantity, and biological activity, which can protect mitochondria from damage. It is particularly essential in the process of cerebral ischemia–reperfusion injury and can protect highly energy‐dependent neurons from the pathological effect of dysfunctional mitochondria [26]. Mitochondrial biogenesis is a procedure by which existing mitochondria produce new mitochondria. This process is generally controlled by PGC‐1 α, which activates NRF1 as well as NRF2 and interacts with them to further activate TFAM, leading to the production of mtDNA and new mitochondria [35]. Meanwhile, it is commonly believed that higher mtDNA copy numbers are beneficial for cells [36]. In this experiment, the intervention of XBP not only facilitated the expressions of PGC‐1α, NRF1 as well as TFAM, but also increased the content of mtDNA, uncovering that XBP promoted mitochondrial biogenesis by increasing PGC‐1α/NRF1/TFAM.

The dynamic characteristics of mitochondria, including fission and fusion, maintain optimal mitochondrial function by controlling morphology, content exchange, fair inheritance of mitochondria, maintaining high‐quality mitochondrial DNA, and isolating damaged mitochondria for degradation [37]. Mitochondrial dynamics are precisely regulated by mitochondrial fusion proteins containing OPA1, MFN1, MFN2, and mitochondrial fission proteins including DRP1, MFF, and FIS1, affecting various aspects of mitochondrial function, especially in the brain where nerve cells have a strong energy demand and are particularly sensitive to the balance of mitochondrial dynamics. Various pieces of evidence suggested that the regulation of mitochondrial dynamics plays an important role in CIR injury, considered a therapeutic target [38, 39]. The results showed that the levels of mitochondrial fission proteins were lessened and that of mitochondrial fusion proteins were increased with XBP treatment. It can be concluded that XBP wields a neuroprotective effect by regulating mitochondrial dynamics. What's more, mitochondrial fission can facilitate mitophagy, which is the process of selectively clearing damaged mitochondria to maintain optimal mitochondrial function. Related studies have shown that CIR is a turning point for mitophagy to transition from a protective to a harmful effect [40]. Our study indicated that XBP could suppress the increase of mitophagy protein expressions in brain injury, revealing that XBP might improve brain injury by inhibiting excessive mitophagy.

AMPK is a key enzyme in regulating cellular energy metabolism and is capable of sensing low ATP levels in cells. Under low‐energy conditions, particular enzymes and growth control nodes can be phosphorylated by AMPK to increase ATP production and reduce ATP consumption [41]. We found that XBP treatment promoted AMPK phosphorylation and increased p‐AMPK expression, suggesting a direct interaction between XBP and AMPK. Increasing studies have illustrated that AMPK can promote mitochondrial health, and multiple literatures have discovered that AMPK targets are involved in various aspects of mitochondrial homeostasis including mitochondrial biogenesis, mitochondrial dynamics, and mitophagy [42, 43]. AMPK has gradually become a crucial regulator for MQC. In this study, we confirmed the regulation of MQC by AMPK mediated XBP against PBI through the intervention of an AMPK inhibitor called Compound C.

Our work has several advancements compared with previous literature. For example, we explored the neuroprotective effect of XBP in PC12 cells and PBI rats. As far as we know, this is the first study to probe the anti‐PBI potential of XBP. However, the limitations should also be recognized. First, our network‐based prediction proposed several important mechanisms related to XBP mitigating CIR, but we only verified the effect of MQC on PBI. Other predicted mechanisms such as inflammation/immune regulation and lipid metabolism are also worth verification in the future. Moreover, we only validated the mitochondria protection via electron microscopy imaging; in vitro and in vivo studies should be further carried out. Finally, we can not accurately assess the actual content of each component in XBP, which should be improved in subsequent experiments, since it is still a difficult problem in the traditional Chinese medicine field.

**FIGURE 11:**
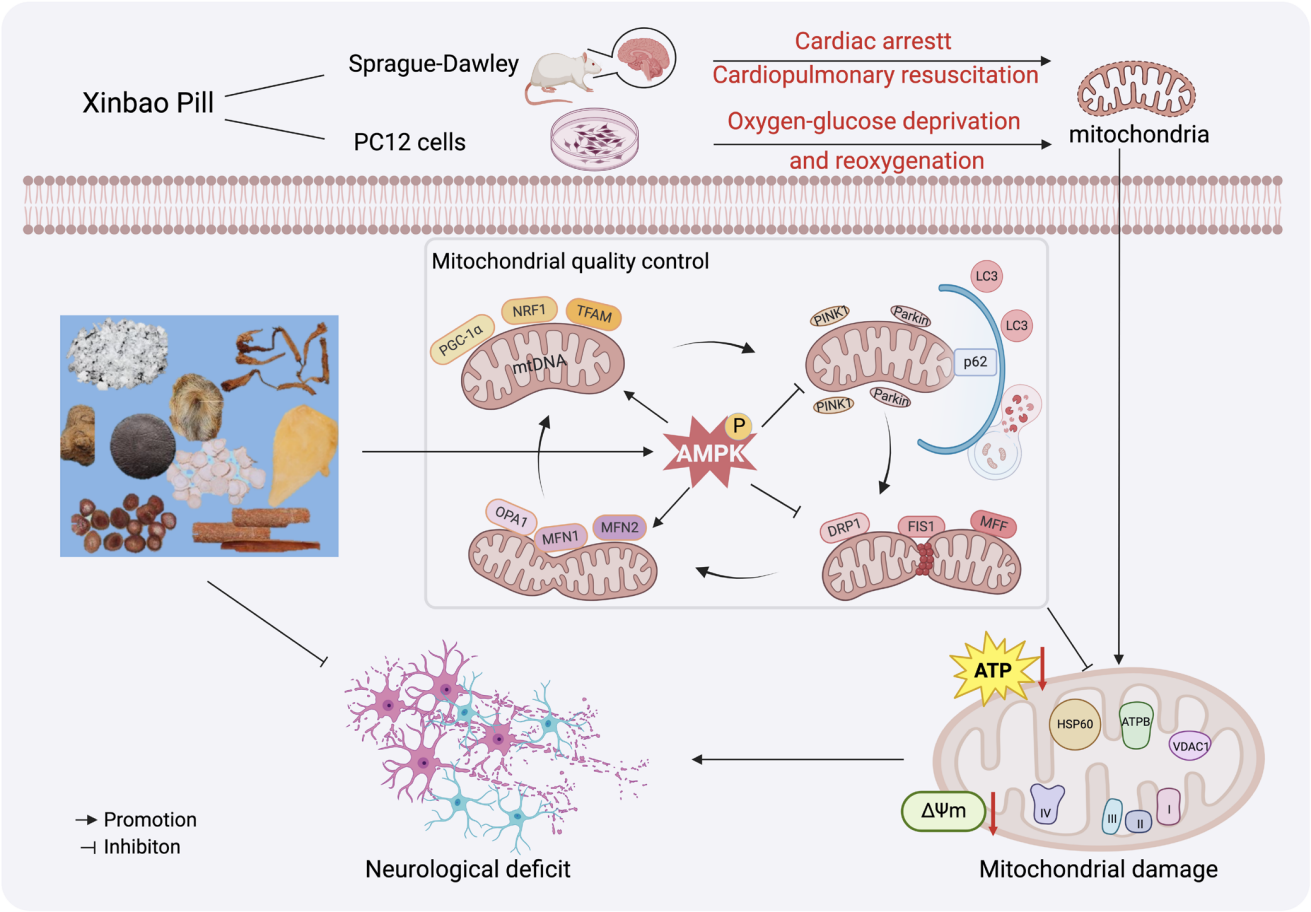
Mechanism diagram of the study. XBP mediated mitochondrial quality control through targeting AMPK, including promoting mitochondrial biogenesis (PGC1α, NRF1, TFAM, mtDNA), mitochondrial fusion (OPA1, MFN1, MFN2) and inhibiting mitochondrial fission (DRP1, MFF, FIS1) as well as excessive mitophagh (p62, PINK1, Parkin, LC3), and further alleviated mitochondrial damage, containing the improvement of ATP, succinate dehydrogenase, mitochondrial respiratory chain complex I ‐ IV, mitochondrial membrane potential, mitochondrial morphology as well as mitochondrial proteins, ultimately exerting neuroprotective effect in PC12 cells under OGD/RO exposure and PBI rats.

This study applied the network pharmacology approach, metabolomics combined with in vivo/in vitro experiments to reveal that XBP mediated MQC to exert a therapeutic effect on PBI through targeting AMPK. The protective role of XBP against CIR injury could be summarized as four aspects, including promoting mitochondrial biogenesis, mitochondrial fusion, and inhibiting mitochondrial fission as well as excessive mitophagy (Figure 11). Overall, this study suggests that XBP may be an alternative drug for the treatment of PBI, which deserves further development and research.

## Author Contributions

B.D. and Y.G. conceived and designed the experiments. D.L. and Q.W. conducted the in vitro experiments and wrote the manuscript. D.L., Z.L., B.C., Q.W., and X.S. conducted the in vivo experiments, and Z.D., L.L., and J.F. contributed to the data collection. R.Z., Y.G., and B.D. reviewed and revised the manuscript. All authors read and approved the final manuscript.

## Conflicts of Interest

The authors declare no conflicts of interest.

## Supporting information


**Figure S1** Drug–target network analysis. Chemical scaffold analysis of the 225 natural products (A) and the structures of each cluster’s center (B). Drug–target network comprises 2644 DTIs interacting with 225 natural products with 1172 genes (C). Label font size and node size are proportional to the degree of connectivity.

**Table S1** 178 genes associated with mitochondrial dysfunction.

## Data Availability

The data that support the findings of this study are available from the corresponding author upon reasonable request.

## References

[1] C. Sandroni , T. Cronberg , and M. Sekhon , “Brain Injury After Cardiac Arrest: Pathophysiology, Treatment, and Prognosis,” Intensive Care Medicine 47, no. 12 (2021): 1393–1414. 34705079 10.1007/s00134-021-06548-2 PMC8548866

[2] G. D. Perkins , C. W. Callaway , K. Haywood , et al., “Brain Injury After Cardiac Arrest,” Lancet 398, no. 10307 (2021): 1269–1278. 34454687 10.1016/S0140-6736(21)00953-3

[3] J. P. Nolan , C. Sandroni , B. W. Bottiger , et al., “European Resuscitation Council and European Society of Intensive Care Medicine Guidelines 2021: Post‐Resuscitation Care,” Intensive Care Medicine 47, no. 4 (2021): 369–421. 33765189 10.1007/s00134-021-06368-4 PMC7993077

[4] H. Uchino , Y. Ogihara , H. Fukui , et al., “Brain Injury Following Cardiac Arrest: Pathophysiology for Neurocritical Care,” Journal of Intensive Care 4 (2016): 31. 27123307 10.1186/s40560-016-0140-9 PMC4847238

[5] H. Benaroya , “Brain Energetics, Mitochondria, and Traumatic Brain Injury,” Reviews in the Neurosciences 31, no. 4 (2020): 363–390. 32004148 10.1515/revneuro-2019-0086

[6] J. Jiang , X. Fang , Y. Fu , et al., “Impaired Cerebral Mitochondrial Oxidative Phosphorylation Function in a Rat Model of Ventricular Fibrillation and Cardiopulmonary Resuscitation,” BioMed Research International 2014 (2014): 192769. 24696844 10.1155/2014/192769 PMC3947758

[7] A. R. Anzell , R. Maizy , K. Przyklenk , and T. H. Sanderson , “Mitochondrial Quality Control and Disease: Insights Into Ischemia‐Reperfusion Injury,” Molecular Neurobiology 55, no. 3 (2018): 2547–2564, 10.1007/s12035-017-0503-9. 28401475 PMC5636654

[8] C. Cardanho‐Ramos and V. A. Morais , “Mitochondrial Biogenesis in Neurons: How and Where,” International Journal of Molecular Sciences 22, no. 23 (2021): 13059, 10.3390/ijms222313059. 34884861 PMC8657637

[9] M. P. C. O. Emergency , L. O. C. I. Key , H. Li , et al., “Expert Consensus for Diagnosis and Treatment of Post‐Cardiac Arrest Syndrome in Adults by Combining Traditional Chinese and Western Medicine in China (2023),” Zhonghua Wei Zhong Bing Ji Jiu Yi Xue 35, no. 10 (2023): 1009–1025. 37873704 10.3760/cma.j.cn121430-20230806-00582

[10] N. Zhao , Y. Gao , H. Jia , and X. Jiang , “Anti‐Apoptosis Effect of Traditional Chinese Medicine in the Treatment of Cerebral Ischemia‐Reperfusion Injury,” Apoptosis 28, no. 5–6 (2023): 702–729. 36892639 10.1007/s10495-023-01824-6

[11] Y. Yang , T. Chen , J. Liu , et al., “Integrated Chemical Profiling, Network Pharmacology and Pharmacological Evaluation to Explore the Potential Mechanism of Xinbao Pill Against Myocardial Ischaemia‐Reperfusion Injury,” Pharmaceutical Biology 60, no. 1 (2022): 255–273, 10.1080/13880209.2022.2025859. 35148221 PMC8845110

[12] Y. Han , X. Li , L. Yang , et al., “Ginsenoside Rg1 Attenuates Cerebral Ischemia‐Reperfusion Injury due to Inhibition of NOX2‐Mediated Calcium Homeostasis Dysregulation in Mice,” Journal of Ginseng Research 46, no. 4 (2022): 515–525, 10.1016/j.jgr.2021.08.001. 35818419 PMC9270650

[13] K. Radad , R. Moldzio , and W. D. Rausch , “Ginsenosides and Their CNS Targets,” CNS Neuroscience & Therapeutics 17, no. 6 (2011): 761–768. 21143430 10.1111/j.1755-5949.2010.00208.x PMC6493809

[14] S. Zhang , Q. Chen , M. Jin , et al., “Notoginsenoside R1 Alleviates Cerebral Ischemia/Reperfusion Injury by Inhibiting the TLR4/MyD88/NF‐KappaB Signaling Pathway Through Microbiota‐Gut‐Brain Axis,” Phytomedicine 128 (2024): 155530. 38493723 10.1016/j.phymed.2024.155530

[15] S. Li , R. Zhang , A. Wang , et al., “Panax Notoginseng: Derived Exosome‐Like Nanoparticles Attenuate Ischemia Reperfusion Injury via Altering Microglia Polarization,” Journal of Nanobiotechnology 21, no. 1 (2023): 416, 10.1186/s12951-023-02161-1. 37946257 PMC10636993

[16] Y. Zhou , X. Zhang , H. Yang , et al., “Mechanism of CAMP Response Element‐Binding Protein 1/Death‐Associated Protein Kinase 1 Axis‐Mediated Hippocampal Neuron Apoptosis in Rat Brain Injury After Cardiopulmonary Resuscitation,” Neuroscience 526 (2023): 175–184, 10.1016/j.neuroscience.2023.06.024. 37406926

[17] X. Tian , Z. Huang , Y. Wang , et al., “Xinbao Pill Attenuated Chronic Heart Failure by Suppressing the Ubiquitination of Beta‐Adrenergic Receptors,” Phytomedicine 115 (2023): 154830. 37149964 10.1016/j.phymed.2023.154830

[18] Y. Tan , J. Zhang , Q. Ge , et al., “Ketone Body Improves Neurological Outcomes After Cardiac Arrest by Inhibiting Mitochondrial Fission in Rats,” Oxidative Medicine and Cellular Longevity 2022 (2022): 7736416. 35847595 10.1155/2022/7736416 PMC9283010

[19] D. Li , C. Cai , Y. Liao , et al., “Systems Pharmacology Approach Uncovers the Therapeutic Mechanism of Medicarpin Against Scopolamine‐Induced Memory Loss,” Phytomedicine 91 (2021): 153662, 10.1016/j.phymed.2021.153662. 34333326

[20] C. G. Fraga , B. H. Clowers , R. J. Moore , and E. M. Zink , “Signature‐Discovery Approach for Sample Matching of a Nerve‐Agent Precursor Using Liquid Chromatography‐Mass Spectrometry, XCMS, and Chemometrics,” Analytical Chemistry 82, no. 10 (2010): 4165–4173, 10.1021/ac1003568. 20405949

[21] Q. Wu , S. Su , C. Cai , et al., “Network Proximity‐Based Computational Pipeline Identifies Drug Candidates for Different Pathological Stages of Alzheimer's Disease,” Computational and Structural Biotechnology Journal 21 (2023): 1907–1920. 36936813 10.1016/j.csbj.2023.02.041 PMC10015208

[22] L. Wang , W. Ren , Q. Wu , et al., “NLRP3 Inflammasome Activation: A Therapeutic Target for Cerebral Ischemia‐Reperfusion Injury,” Frontiers in Molecular Neuroscience 15 (2022): 847440, 10.3389/fnmol.2022.847440. 35600078 PMC9122020

[23] M. Franke , M. Bieber , P. Kraft , A. N. R. Weber , G. Stoll , and M. K. Schuhmann , “The NLRP3 Inflammasome Drives Inflammation in Ischemia/Reperfusion Injury After Transient Middle Cerebral Artery Occlusion in Mice,” Brain, Behavior, and Immunity 92 (2021): 223–233, 10.1016/j.bbi.2020.12.009. 33307174

[24] Z. He , B. K. Yin , K. Wang , et al., “The Alpha2‐Adrenergic Receptor Agonist Clonidine Protects Against Cerebral Ischemia/Reperfusion Induced Neuronal Apoptosis in Rats,” Metabolic Brain Disease 39, no. 5 (2024): 741–752, 10.1007/s11011-024-01354-3. 38833094

[25] U. Yilmaz , K. Tanbek , S. Gul , A. Koc , M. Gul , and S. Sandal , “Intracerebroventricular BDNF Infusion May Reduce Cerebral Ischemia/Reperfusion Injury by Promoting Autophagy and Suppressing Apoptosis,” Journal of Cellular and Molecular Medicine 28, no. 8 (2024): e18246. 38520223 10.1111/jcmm.18246 PMC10960178

[26] M. Wu , X. Gu , and Z. Ma , “Mitochondrial Quality Control in Cerebral Ischemia‐Reperfusion Injury,” Molecular Neurobiology 58, no. 10 (2021): 5253–5271. 34275087 10.1007/s12035-021-02494-8

[27] Z. Ma , Z. Xin , W. Di , et al., “Melatonin and Mitochondrial Function During Ischemia/Reperfusion Injury,” Cellular and Molecular Life Sciences 74, no. 21 (2017): 3989–3998. 28795196 10.1007/s00018-017-2618-6 PMC11107672

[28] J. K. Shin and S. M. Lee , “Genipin Protects the Liver From Ischemia/Reperfusion Injury by Modulating Mitochondrial Quality Control,” Toxicology and Applied Pharmacology 328 (2017): 25–33. 28477916 10.1016/j.taap.2017.05.002

[29] Y. Zhang , Y. He , M. Wu , et al., “Rehmapicroside Ameliorates Cerebral Ischemia‐Reperfusion Injury via Attenuating Peroxynitrite‐Mediated Mitophagy Activation,” Free Radical Biology & Medicine 160 (2020): 526–539. 32784031 10.1016/j.freeradbiomed.2020.06.034

[30] W. C. Xiao , G. Zhou , L. Wan , et al., “Carnosol Inhibits Cerebral Ischemia‐Reperfusion Injury by Promoting AMPK Activation,” Brain Research Bulletin 195 (2023): 37–46. 36775042 10.1016/j.brainresbull.2023.02.003

[31] J. Zhou , F. Sun , W. Zhang , Z. Feng , Y. Yang , and Z. Mei , “Novel Insight Into the Therapeutical Potential of Flavonoids From Traditional Chinese Medicine Against Cerebral Ischemia/Reperfusion Injury,” Frontiers in Pharmacology 15 (2024): 1352760, 10.3389/fphar.2024.1352760. 38487170 PMC10937431

[32] T. Zheng , T. Jiang , Z. Huang , H. Ma , and M. Wang , “Role of Traditional Chinese Medicine Monomers in Cerebral Ischemia/Reperfusion Injury: A Review of the Mechanism,” Frontiers in Pharmacology 14 (2023): 1220862, 10.3389/fphar.2023.1220862. 37654609 PMC10467294

[33] S. S. Kumar and D. Singh , “Mitochondrial Mechanisms in Cerebral Ischemia‐Reperfusion Injury: Unravelling the Intricacies,” Mitochondrion 77 (2024): 101883. 38631511 10.1016/j.mito.2024.101883

[34] F. Han , T. Da , N. A. Riobo , et al., “Early Mitochondrial Dysfunction in Electron Transfer Activity and Reactive Oxygen Species Generation After Cardiac Arrest,” Critical Care Medicine 36 (2008): S447–S453. 20449909 10.1097/ccm.0b013e31818a8a51 PMC3315374

[35] P. A. Li , X. Hou , and S. Hao , “Mitochondrial Biogenesis in Neurodegeneration,” Journal of Neuroscience Research 95, no. 10 (2017): 2025–2029. 28301064 10.1002/jnr.24042

[36] L. D. Popov , “Mitochondrial Biogenesis: An Update,” Journal of Cellular and Molecular Medicine 24, no. 9 (2020): 4892–4899. 32279443 10.1111/jcmm.15194 PMC7205802

[37] D. C. Chan , “Mitochondrial Dynamics and Its Involvement in Disease,” Annual Review of Pathology 15 (2020): 235–259. 10.1146/annurev-pathmechdis-012419-032711 31585519

[38] H. Wang , S. Chen , Y. Zhang , H. Xu , and H. Sun , “Electroacupuncture Ameliorates Neuronal Injury by Pink1/Parkin‐Mediated Mitophagy Clearance in Cerebral Ischemia‐Reperfusion,” Nitric Oxide 91 (2019): 23–34, 10.1016/j.niox.2019.07.004. 31323277

[39] J. Vongsfak , W. Pratchayasakul , N. Apaijai , T. Vaniyapong , N. Chattipakorn , and S. C. Chattipakorn , “The Alterations in Mitochondrial Dynamics Following Cerebral Ischemia/Reperfusion Injury,” Antioxidants 10, no. 9 (2021): 1384, 10.3390/antiox10091384. 34573016 PMC8468543

[40] Z. Li and J. Xing , “Contribution and Therapeutic Value of Mitophagy in Cerebral Ischemia‐Reperfusion Injury After Cardiac Arrest,” Biomedicine & Pharmacotherapy 167 (2023): 115492. 37716121 10.1016/j.biopha.2023.115492

[41] S. Herzig and R. J. Shaw , “AMPK: Guardian of Metabolism and Mitochondrial Homeostasis,” Nature Reviews. Molecular Cell Biology 19, no. 2 (2018): 121–135. 28974774 10.1038/nrm.2017.95 PMC5780224

[42] A. P. Seabright , N. Fine , J. P. Barlow , et al., “AMPK Activation Induces Mitophagy and Promotes Mitochondrial Fission While Activating TBK1 in a PINK1‐Parkin Independent Manner,” FASEB Journal 34, no. 5 (2020): 6284–6301, 10.1096/fj.201903051r. 32201986 PMC7212019

[43] T. L. Marin , B. Gongol , F. Zhang , et al., “AMPK Promotes Mitochondrial Biogenesis and Function by Phosphorylating the Epigenetic Factors DNMT1, RBBP7, and HAT1,” Science Signaling 10, no. 464 (2017): eaaf7478, 10.1126/scisignal.aaf7478. 28143904 PMC5830108

