# Supplementary figures and images for: Exploring the Therapeutic Mechanism of Xinbao Pill in Brain Injury After Cardiopulmonary Resuscitation Based on Network Pharmacology, Metabolomics, and Experimental Verification
Source: CNS Neurosci Ther. 2025 Mar 4;31(3):e70297. doi: 10.1111/cns.70297 (PMC11877134; doi:10.1111/cns.70297)

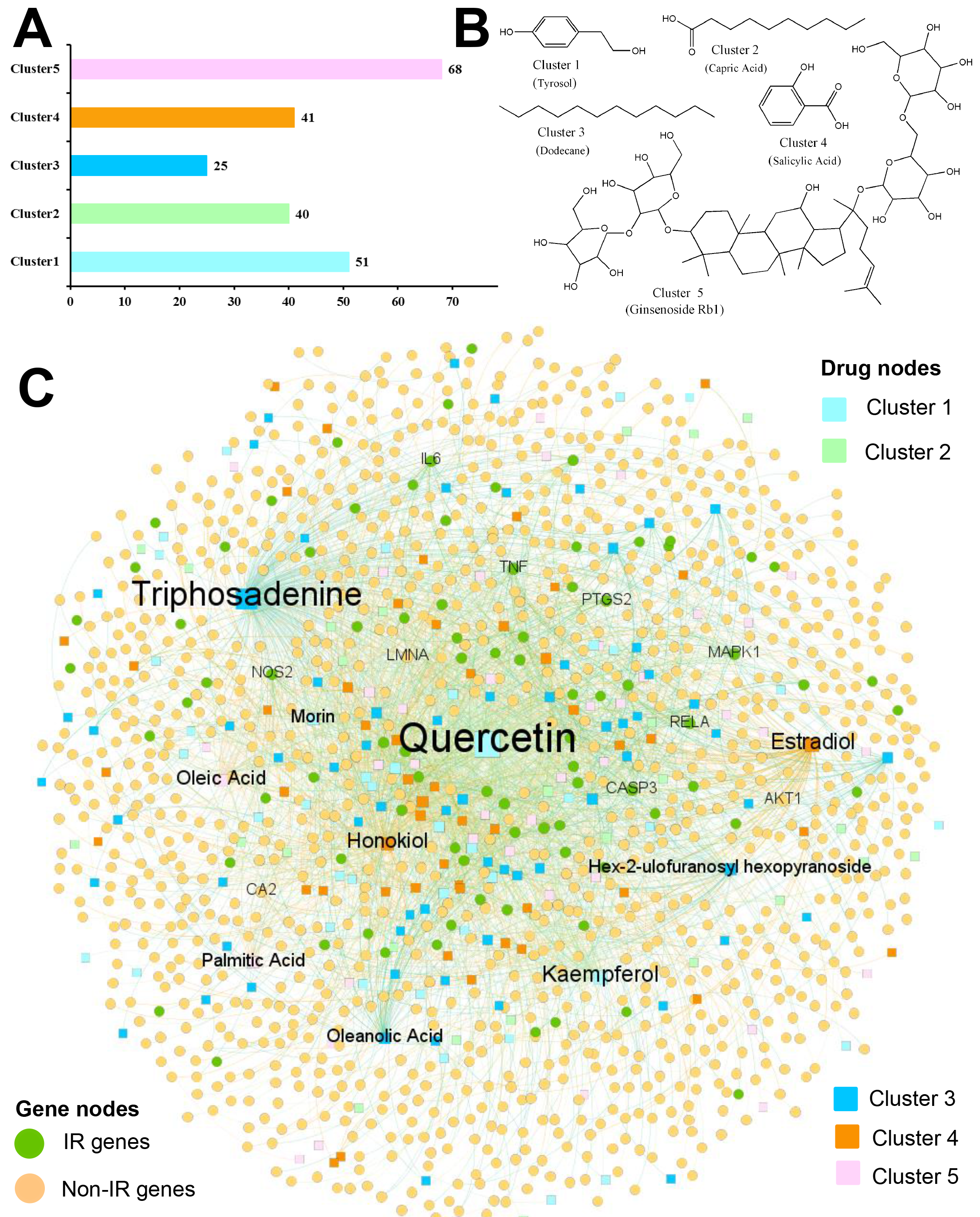

Supplement: Supplementary file 1 — Figure S1 Drug–target network analysis. Chemical scaffold analysis of the 225 natural products (A) and the structures of each cluster’s center (B). Drug–target network comprises 2644 DTIs interacting with 225 natural products with 1172 genes (C). Label font size and node size are proportional to the degree of connectivity. [file CNS-31-e70297-s002.tif]
